# Border cells without theta rhythmicity in the medial prefrontal cortex

**DOI:** 10.1073/pnas.2321614121

**Published:** 2024-06-10

**Authors:** Xiaoyang Long, Bin Deng, Rui Shen, Lin Yang, Liping Chen, Qingxia Ran, Xin Du, Sheng-Jia Zhang

**Affiliations:** ^a^Department of Neurosurgery, Xinqiao Hospital, Army Medical University, Chongqing 400037, China

**Keywords:** medial prefrontal cortex, border cell, theta rhythmicity, allocentric and egocentric coordinate, spatial navigation

## Abstract

The medial prefrontal cortex (mPFC) is a key brain structure for higher cognitive functions such as decision-making and goal-directed behavior, many of which require awareness of spatial variables including one’s current position within the surrounding environment. Although previous studies have reported spatially tuned activities in mPFC during memory-related trajectory, the spatial tuning of mPFC network during freely foraging behavior remains elusive. Here, we reveal geometric border or border-proximal representations from the neural activity of mPFC ensembles during naturally exploring behavior, with both allocentric and egocentric boundary responses. Unlike most of classical border cells in the medial entorhinal cortex (MEC) discharging along a single wall, a large majority of border cells in mPFC fire particularly along four walls. mPFC border cells generate new firing fields to external insert, and remain stable under darkness, across distinct shapes, and in novel environments. In contrast to hippocampal theta entrainment during spatial working memory tasks, mPFC border cells rarely exhibited theta rhythmicity during spontaneous locomotion behavior. These findings reveal spatially modulated activity in mPFC, supporting local computation for cognitive functions involving spatial context and contributing to a broad spatial tuning property of cortical circuits.

## Significance

Spatial navigation is a complex cognitive function that requires the coordination of multiple brain regions, which critically involves the interplay between the medial prefrontal cortex (mPFC) and the hippocampus. Despite the widely recognized role of the hippocampus in the formation and retrieval of spatial memory, the spatial coding properties of mPFC neurons during naturalistic behaviors remain elusive. Here, we report the identification of a type of mPFC border cells in freely moving rats, which show low theta rhythmicity and display both allocentric and egocentric spatial tuning. These border cells might support the local mPFC neural circuit for the execution of spatial memory–related tasks and suggest that spatial representation in the brain is more widely distributed than previously thought.

The ventral part of the medial prefrontal cortex (mPFC) contains prelimbic cortex and infralimbic cortex (1, 2), and is reciprocally connected with the anterior cingulate cortex (ACC) (3). The mPFC has been suggested to be involved in many higher cognitive behaviors such as planning, attention, goal-directed, social, and emotional behaviors (1, 4, 5), and many of these processes involve the awareness of the spatial information of one’s own position within the surrounding environments.

The interplay between mPFC and hippocampus has been implicated in coordinating various behavioral and cognitive functions (6, 7). A direct pathway from the hippocampus to mPFC originates from the ventral hippocampal CA1 area and the subiculum (8, 9). Additionally, mPFC also receives inputs from deep layers of medial entorhinal cortex (10). The hippocampal and parahippocampal network has been the frontier in dissecting the cellular and circuit mechanisms of spatial representation in the brain and is implicated in mediating spatial navigation and memory (11). Cells recorded from the CA1 region of the hippocampus are reminiscent of their location-specific firing patterns in the environment, known as place cells (12), while the subiculum and MEC deep layer exhibit a variety of spatial responses from grid cells, border cells, and head direction cells (13–18). Thus, the possibility arises that the prefrontal recipients of hippocampal efferent may also display spatial firing patterns. Moreover, lesion studies in rodents have implicated the involvement of mPFC in multiple aspects of spatial navigation including spatial memory encoding and retrieval (19–21), and different prefrontal regions might contribute to distinct stages of spatial navigation (22).

Spatially tuned activities in the mPFC have been reported during cognitive tasks such as goal-directed behavior (23–27). However, this behavior paradigm involves active rule learning, and requires the animal to learn the reward location, which is different from the freely foraging paradigm typically used in identifying spatial cells. The search for spatial cells within the mPFC in freely foraging animals was examined in several previous studies (23, 27–31). Earlier recordings from the prelimbic area revealed that the firing field of certain cells was either a large circular region almost centered in the cylinder or, on the contrary, a large annulus-shaped, peripheral, region of the cylinder (28). Interestingly, mPFC neurons exhibited spatial correlates during a place navigation task (31), indicating a role of mPFC for spatial coding in navigation (30). Additionally, annulus cells were identified in the ACC with peak firing rates around the periphery of the environment (32). The ACC and ventral mPFC are closely interconnected and show similar recruitments in a multitude of cognitive tasks (33). Furthermore, a recent study reported a topographically organized representation of spatial location and context in the dorsal–ventral axis of mPFC when mice were navigating in a virtual environment in a task- and rule-free manner (34). Altogether, these results suggested the existence of spatially tuned activities in mPFC.

To test whether mPFC neurons are spatially tuned during freely foraging behavior, we made chronic recordings from prelimbic and infralimbic cortices of mPFC. We found that a subset of mPFC neurons (11.19%) exhibited spatially tuned firing patterns to geometric borders. These spatial responses were stable and did not remap across distinct environments. Border representations were retained under darkness and were contingent on physical boundaries with new firing fields introduced by external insert. Further classification of mPFC border cells revealed both allocentric and egocentric tuning properties, with clear lateralization of egocentric responses. In addition, mPFC border cells were rarely theta-rhythmic, indicative of independence from hippocampal entrainment. These results contrast with previous findings that no apparent spatial tuning properties were observed in mPFC neurons during naturally exploring behaviors (27, 29). Altogether, our findings support the view of wide spatial representations across cortical regions, and the spatial tuning of mPFC might be relevant for processing local spatial information to support higher cognitive functions.

## Results

### Extracellular Recordings in mPFC from Freely Foraging Rats.

**Fig. 1.**
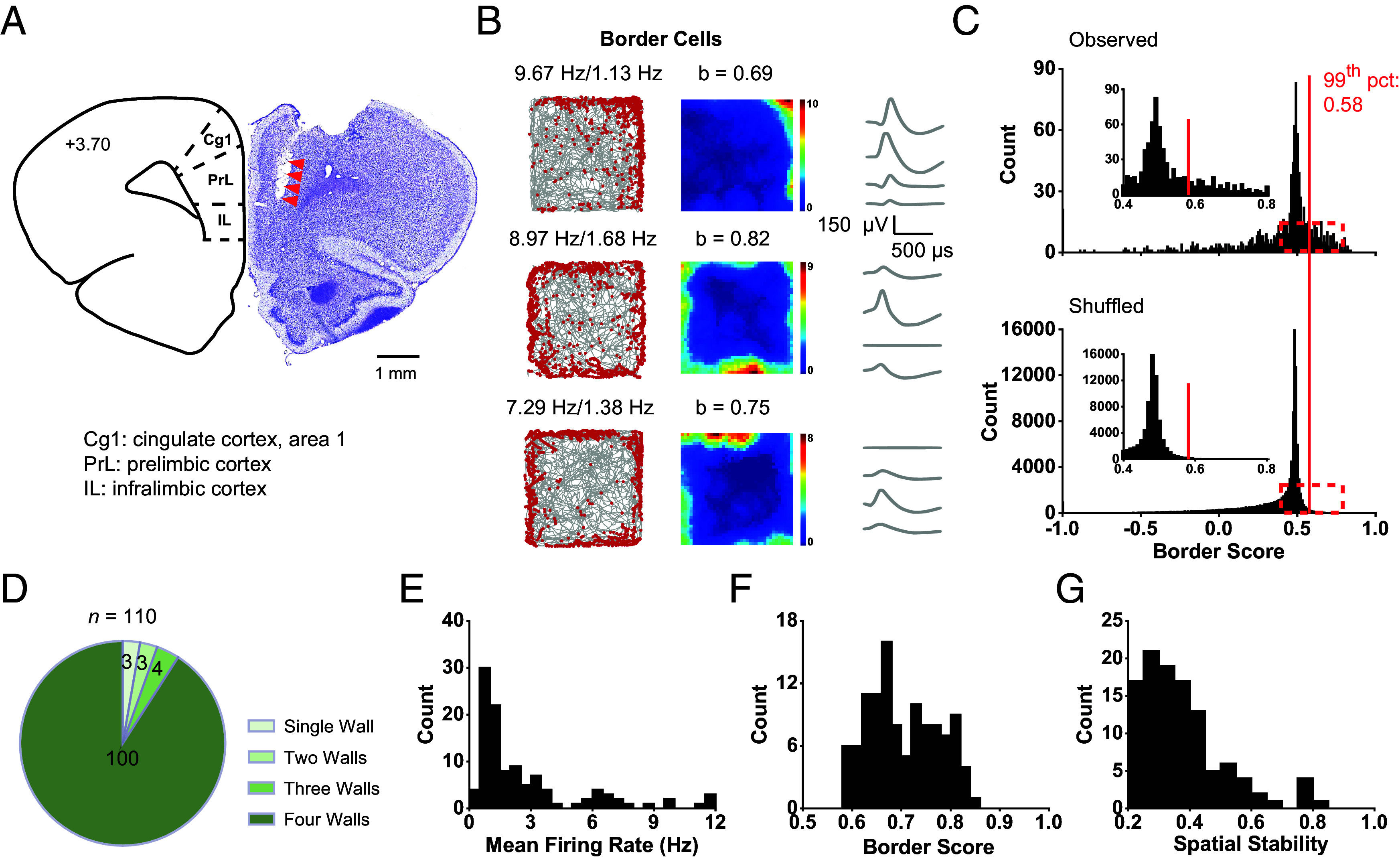
Geometric border representations in mPFC. (*A*) Nissl staining showing the anatomical location of the electrode tracks in mPFC. The red arrow indicates the electrode tracks. (Scale bar, 1 mm.) (*B*) Three representative border cells in mPFC. *Left*, trajectory (gray line) with superimposed spike locations (red dots); *Middle*, firing rate maps; *Right*, spike waveforms on four tetrodes. Peak firing rate, mean firing rate, and border score were labeled at the *Top* of the panels. (Scale bar, 500 μs and 150 μV.) (*C*) Distribution of border score for observed data (*Top*) and shuffled data (*Bottom*). The red line indicates the 99th percentile for the border score derived from the shuffled data. The *Inset* shows the zoomed-in of the red dashed box. (*D*) Pie chart showing the ratio of border cells with firing field along one, two, three, or four walls. (*E*–*G*) Histograms showing the distribution of mean firing rate, border score, and spatial stability for all identified mPFC border cells.

We recorded neuronal activity from the left or right hemisphere of mPFC of implanted male Long-Evans adult rats (*n* = 10) using microdrives with movable tetrodes (*SI Appendix*, Table S1), as rats foraged freely in a 1 × 1 m^2^ open square arena with 50 cm-high walls. Reconstruction of the electrode location using Nissl staining combined with electrolytic lesions confirmed the recording sites from the ventral part of the mPFC, which is composed of prelimbic (PrL) and infralimbic cortices (IL) (Fig. 1*A* and *SI Appendix*, Fig. S1). In total, we obtained 983 well-isolated single units (*SI Appendix*, Fig. S2), mainly from the deep layer V/VI of mPFC.

### Geometric Border Representations in mPFC.

We found that spatial tuning of geometric borders emerged in the mPFC during freely moving recordings (Fig. 1*B*). Border cells increase their firing rates when the animal is close to the geometric borders of the environment (13, 17). An mPFC unit was defined as a border cell if both its border score (Fig. 1*C*) and spatial stability (*SI Appendix*, Fig. S3) were greater than the corresponding 99th percentile of shuffled population data. The classifying threshold for border score (0.58) was similar to those reported in presubiculum (0.57), parasubiculum (0.58), and MEC (0.57) (16). In total, 110 out of 983 (11.19%) units passed the classification threshold and were classified as border cells. This percentage was significantly higher than expected by random selection from the entire shuffled population (*Z* = 32.11, *P* < 0.001; binomial test with expected *P*_0_ of 0.01 among large samples), indicating that the observed spatial tuning of mPFC neurons was not due to chance. Notably, a large majority (100/110, 90.91%) of mPFC border cells increased their firing rates along four boundaries of the environment (Fig. 1*D*). This is in contrast to the dominance of firing fields along a single wall of MEC border cells (13), where 52 out of 69 (75.36%) border cells fired along a single wall and the remaining 17 from the deep layers of MEC, had firing fields along two, three, or four walls. Further investigations of the superficial layers II/III of the mPFC will help elucidate whether the differences between mPFC and MEC were layer-dependent.

To avoid complex behaviors such as grooming or rearing, which are known to modulate the activity of prefrontal neurons (29), we filtered spike activity only with running speed greater than 2.5 cm/s and found that border score was significantly higher during active running than immobility (*SI Appendix*, Fig. S4 *A* and *B*), with mean firing rate also being slightly higher (*SI Appendix*, Fig. S4*C*). To investigate whether the spatial representations of mPFC border cells resulted from more occupancy at geometric boundaries, we calculated the correlation between the firing rate maps and the occupancy time maps (*SI Appendix*, Fig. S5*A*) and found that the correlation coefficients were generally low and the border score did not positively correlate with the correlation coefficients (Pearson’s *r* = –0.14, *P* = 0.15; *SI Appendix*, Fig. S5 *B* and *C*). Therefore, spatial representations of mPFC border cells were not artifacts from more occupancy at surrounding geometric boundaries.

The firing of mPFC cells has been known to exhibit reward-related modulation (31, 35–38). To investigate the effect of reward on the spatial firing properties of mPFC border cells, we first calculated the heat maps of cumulative density of food pellets and found there was no significant accumulation of food near the periphery of the environment (*SI Appendix*, Fig. S6). Next, we found the responses of mPFC border cells with or without throwing food pellets into the running arena preserved, with both border score and mean firing rate remained unchanged (*SI Appendix*, Fig. S7). Finally, we recorded mPFC border cells in a goal-oriented task with food pellets delivered into a fixed location only and the reward zone was shifted between two recording sessions. We did not observe an increase in firing rate in the reward zone compared to that in the baseline session or compared to that near the boundary of the running arena (*SI Appendix*, Fig. S8). Meanwhile, consistent with previous studies, we observe a similar proportion mPFC cells (7/35, *SI Appendix*, Fig. S8*C*) responded in a space-locked manner to the reward location with increased firing rate in the reward zone (*SI Appendix*, Fig. S8 *B* and *F*). Thus, border cells and reward-related cells coexist in mPFC, and the characterization of the spatial and behavioral correlates of the neuronal population within mPFC requires further investigation.

Based on mean firing rate and spike waveform kinetics, we grouped identified cells into putative pyramidal excitatory cells and GABAergic inhibitory interneurons (*SI Appendix*, Fig. S9 *A* and *B*). We classified putative fast-spiking interneurons as cells with an average firing rate above 5 Hz and a peak-to-trough spike width below 300 µs, based on previous studies (39–41). According to this criterion, 68.18% (*n* = 75/110) of recorded border cells were classified as regular-spiking (RS) cells, 6.36% (*n* = 7/110) were classified as fast-spiking (FS) cells, and 25.45% (*n* = 28/110) were unclassified cells (*SI Appendix*, Fig. S9 *C* and *D*). Despite their differences in electrophysiological properties, these three groups of border cells exhibited similar border scores [one-way ANOVA test, F(3) = 1.47, *P* = 0.24, post hoc Bonferroni multiple comparisons test, RS versus FS: *P* = 0.44; FS versus Unclassified: *P* = 0.27; RS versus Unclassified: *P* = 1.00; *SI Appendix*, Fig. S9*E*].

### Coexistence of Allocentric and Egocentric Border Cells in mPFC.

**Fig. 2.**
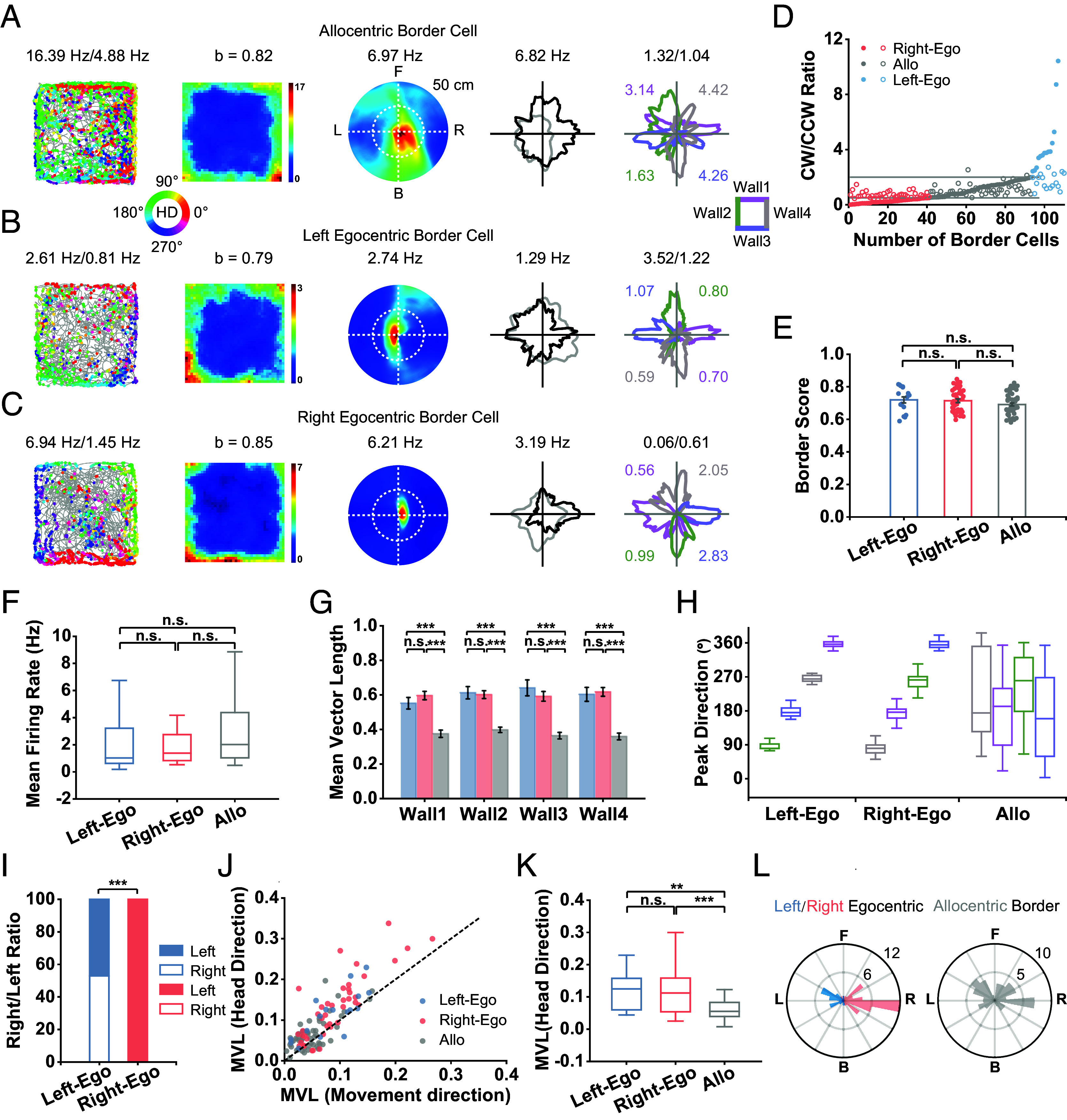
Coexistence of allocentric and egocentric responses in mPFC. (*A*–*C*) Representative allocentric (*A*), left egocentric (*B*), and right egocentric (*C*) border cells. From *Left* to *Right*, color-coded cross-trajectory (gray line) with superimposed directional spike locations (color circles indicate the corresponding head direction; color bar shows the directional range 0° to 360°) (1st column); heat maps of spatial firing rate (2nd column); EBRs (3rd column); head-direction tuning curves (black) plotted against dwell-time polar plot (gray) (4th column); head-direction tuning curves for the corresponding color-coded four walls (right insert) with peak firing rates labeled (5th column). The firing rate was color-coded with dark blue (red) indicating the minimal (maximal) firing rate. Peak firing rate (fr), mean firing rate, border score (b), peak angular rate, the ratios of firing rate and total time between animal’s heading in a clockwise direction and counterclockwise directions (fr_cw/ccw and time_cw/ccw), for each border cell are labeled at the *Top* of the panels. (*D*) The distribution of time_cw/ccw (open dot) and fr_cw/ccw (solid dot) for three subtypes of border cells. (*E*) The border score of three subtypes of border cells. All data are represented as mean ± SEM unless stated otherwise. (*F*) The mean firing rate of three subtypes of border cells. (*G*) Comparison of mean vector length calculated for four separate walls across three subtypes of border cells. (*H*) The summary of peak directions of head-direction tuning for three subtypes of border cells. (*I*) The ratio of left egocentric cells and right egocentric border cells recorded in the left versus right hemisphere. (*J*) Scatterplot of mean vector lengths calculated using movement direction as reference angle versus using head direction as reference angle for left/right egocentric border cells and allocentric border cells. (*K*) Comparison of mean vector length of egocentric tuning among left egocentric, right egocentric, and allocentric border cells. (*L*) Rose diagrams showing the preferred angle of EBRs for left and right egocentric border cells (*Left*) and allocentric border cells (*Right*).

To further refine the tuning properties, we sorted mPFC border cells into allocentric or egocentric border cells based on whether a cell increases its firing rate when the animal approaches the geometric borders in a bidirectional manner or unidirectional manner. A cell was defined as an allocentric border cell if the cell exhibited symmetric firing patterns in either direction next to the geometric border (Fig. 2*A*). Conversely, if the cell exhibited a significant asymmetric directional firing manner, the cell was defined as an egocentric border cell. Specifically, if a cell only increases firing when the environmental boundary was on the left or right side, the cell was defined as a left (Fig. 2*B*) or right (Fig. 2*C*) egocentric border cell.

To classify allocentric or egocentric border cells, we calculated the ratio of each neuron’s firing rate along the geometric borders during clockwise (CW) versus counterclockwise (CCW) heading. If the ratio was between 0.5 and 2, then the border cell was classified as an allocentric border cell; in contrast, if the ratio was greater than 2, the border cell was classified as a left egocentric border cell; if the ratio was less than 0.5, the border cell was classified as a right egocentric border cell. Accordingly, 18.18% (20/110) of identified mPFC border cells were classified as left egocentric border cells, 35.45% (39/110) of identified mPFC border cells were classified as right egocentric border cells, and 46.36% (51/110) were classified as allocentric border cells (Fig. 2*D*). There were no significant differences in either border score [Fig. 2*E*, one-way ANOVA test, F(3) = 2.53, *P* = 0.08, post hoc Bonferroni multiple comparisons test, *P* > 0.05] or mean firing rate [Fig. 2*F*, one-way ANOVA test, F(3) = 0.99, *P* = 0.38, post hoc Bonferroni multiple comparisons test, *P* > 0.05] among left egocentric, right egocentric, and allocentric border cells.

To quantify the selectivity of directional tuning of the three subtypes of border cells, we calculated the mean vector lengths along four individual walls of the arena. Since sampling of directions perpendicular to the wall is very sparse, the firing direction along the wall was mainly parallel to the wall. For egocentric border cells, the directional tuning along each single wall was unipolar in one direction which led to a high mean vector length. However, for allocentric border cells, the directional selectivity along each wall canceled out with two directions opposite to each other which in turn resulted in low mean vector length. Left and right egocentric border cells showed strong tuning to the geometric walls, exhibiting higher mean vector length than allocentric border cells (Fig. 2*G*, one-way ANOVA test, all with the post hoc Bonferroni multiple comparisons test, n.s., *P* > 0.05; ***P < 0.001). The summary of preferred directions in the head-direction tuning of egocentric border cells displayed a 90° step increase along four walls (Fig. 2*H*). In contrast, allocentric border cells exhibited similar peak directions for two opposite walls (Fig. 2*H*) as a consequence of bidirectional firing during CW and CCW rotations along the geometric boundaries.

There was a clear distribution bias between the left and right hemispheres. Remarkably, 100% (41/41) of right egocentric border cells were recorded from the left hemisphere, whereas 52.94% (9/17) of left egocentric border cells were located in the right hemisphere (Fig. 2*I*). The ratios of left and right egocentric border cells recorded in the right versus the left hemisphere were significantly different (Fig. 2*I*, chi-squared test, *P* < 0.001). This lateralization distribution, i.e., the tuning direction is contralateral with the hemisphere where the neuron is located, may offer clues to the sensory origin of the mPFC egocentric signals. However, since 91 border-proximal cells from the left hemisphere and the rest 19 border-proximal cells from the right hemisphere were recorded (*SI Appendix*, Table S1), the 100% right egocentric cells recorded from the left hemisphere might be due to the large asymmetric sampling.

To further verify the egocentric tuning property, we constructed egocentric boundary ratemaps (EBRs) using methods previously described (42, 43). EBRs are used to determine the orientation and distance of the geometric boundaries relative to the animal’s head direction when a cell fires. Briefly, for each video frame, the intersection from the animals’ current location and heading to the nearest geometric borders in steps of 3° across 360° and distance binned at 2.5 cm was calculated. The procedure was repeated for each spike emitted by a given cell and each time sample the animal has occupied, and by dividing the number of spikes by the amount of occupancy, an EBR in polar coordinates was constructed. Specifically, in terms of distance, when a cell fires at the center, it means the cell fires along the geometric borders. In terms of orientation, if a cell fires at 0° or 180°, it means that the borders are located in front or back of the animal, respectively. On the other hand, 90° or 270° means the borders are located on its left or right, respectively (*Middle* panels in Fig. 2 *A*–*C*).

We calculated the mean vector length for each EBR to measure the strength of egocentric tuning and the preferred angular tuning direction of each recorded border cell. While both head direction and movement direction could be used as the orientation reference to construct egocentric receptive fields, the strength of egocentric tuning to head direction was stronger than that to movement direction (Fig. 2*J*). Moreover, the mean vector length of the left and right egocentric border cells was significantly higher than that of allocentric border cells [Fig. 2*K*, one-way ANOVA test, F(3) = 11.03, *P* < 0.001, post hoc Bonferroni multiple comparisons test, Left-Ego versus Right-Ego: *P* = 1.00; Right-Ego versus Allo: *P* <0.001; Left-Ego versus Allo: *P* = 0.03]. The preferred angular tuning direction was defined as the mean direction of the mean vector of the EBR for each recorded border cell. The preferred angular tuning direction was clustered toward the left and right side of the animal for left and right egocentric border cells, respectively, and toward both sides for allocentric border cells (Fig. 2*L*).

### mPFC Border Cells Respond to New Insert.

**Fig. 3.**
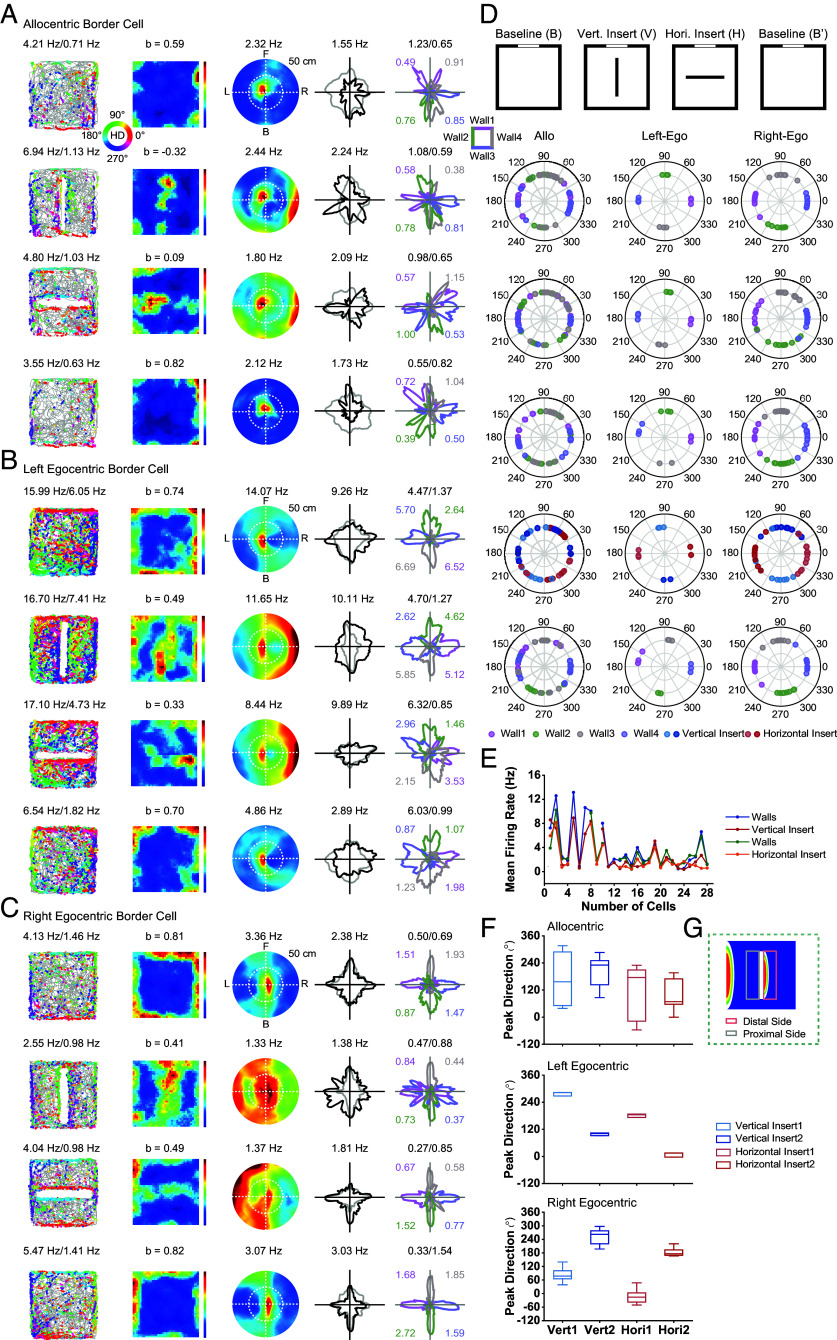
mPFC border cells respond to new insert. (*A*–*C*) Spatial responses of representative allocentric (*A*), left egocentric (*B*), and right egocentric (*C*) border cells, respectively, to the introduction of new insert into the environments. Notations and symbols are similar to Fig. 2. (*D*) *Top*, diagram of the experimental procedure with the baseline, inserted vertical wall, inserted horizontal wall, and second baseline conditions. *Bottom*, polar plot showing the distribution of peak direction of head-direction tuning along color-coded walls and new inserts. From *Top* to *Bottom*, preferred firing directions along four walls in the baseline session, in the vertical insert session, in the horizontal insert session, along both sides of vertical and horizontal inserts, and along four walls in another baseline session. (*E*) Comparison of mean firing rate along four walls and two boundaries of the new insert for both vertical insert and horizontal insert conditions. (*F*) Summary of peak directions along the two sides of the new insert for left egocentric, right egocentric, and allocentric border cells, respectively. The left and right egocentric border cells exhibited opposite directional tuning along the same side of the insert, while allocentric border cells showed both two opposite directional tuning along the same side of the insert. (*G*) Diagram showing the definition of the proximal and distal sides along the insert relative to one specific parallel wall.

To verify whether mPFC border cells respond to general borders instead of environmental walls, we inserted a discrete wall into the open area creating two additional borders besides four walls of the environment. We recorded the activity of 14 allocentric border cells, 3 left egocentric border cells, and 11 right egocentric border cells in environments with new insert (Fig. 3 *A*–*C*). The proximal and distal sides of the insert were defined as a 15-cm wide rectangle along the insert relative to one specific parallel wall (Fig. 3*G*). This manipulation resulted in new border fields on both proximal and distal sides of the external insert of both allocentric and egocentric border cells, with comparable mean firing rates along the four walls and the new insert (Fig. 3*E*).

Notably, the newly evoked firing fields along the new insert of egocentric border cells displayed parallel head-direction tuning to those of the corresponding distal walls (Fig. 3*D*), while those of allocentric border cells showed bidirectional firing along the new insert similar to the four walls of the square enclosure (Fig. 3*D*). Specifically, for left and right egocentric border cells, the preferred directions of newly evoked border fields on the same side of the new insert were opposite. Together, left and right egocentric border cells showed opposite unidirectional tuning along the same side of the new insert, and their peak directions were nearly 180° apart (Fig. 3*F*), while allocentric border cells showed bidirectional tuning along the same side of the new insert (Fig. 3*F*).

### mPFC Border Cells Respond to Small Objects.

**Fig. 4.**
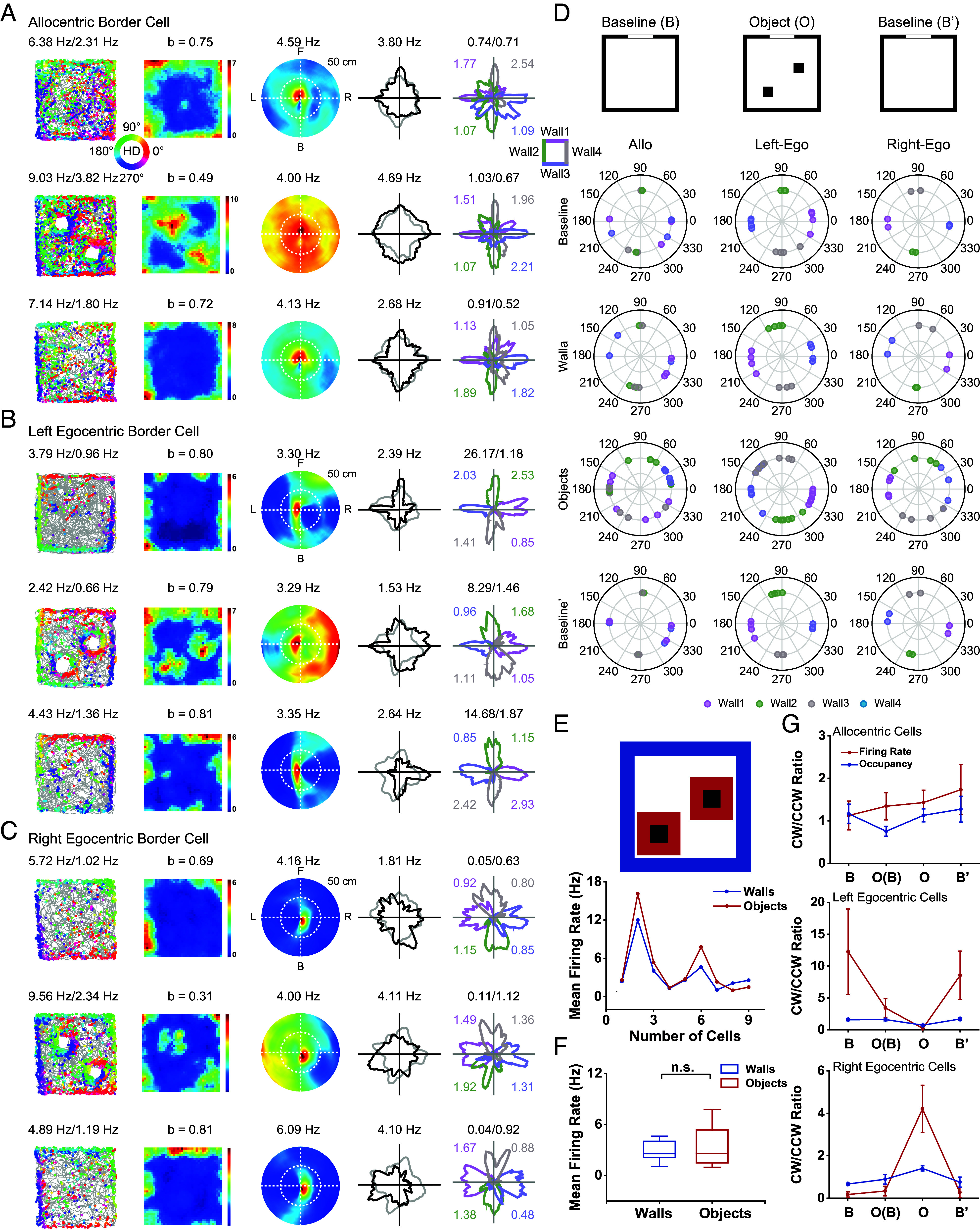
Responses of mPFC border cells in the presence of small objects. (*A*–*C*) Spatial responses of representative allocentric (*A*), left egocentric (*B*), and right egocentric (*C*) border cells, respectively, in the baseline (B), in the presence of objects (O), and in another baseline (B’) sessions. Notations and symbols are similar to those in Fig. 2. (*D*) Distributions of peak directions along the four sides of the walls and objects for allocentric, left egocentric, and right egocentric border cells, respectively. From *Top* to *Bottom*, preferred firing directions along four walls in the baseline session, in object session, along four sides of the objects, and along four walls in another baseline session. (*E*) *Top*, diagram showing the boundaries along the four sides of the walls (blue) and the objects (red). *Bottom*, scatterplot showing the mean firing rate along the walls and the objects. (*F*) Mean firing rate along the walls and the objects did not exhibit significant differences. (*G*) Left and right egocentric border cells kept CW/CCW firing rate ratio along the boundaries of new objects placed in the environment.

Furthermore, to test whether egocentric border cells respond to small objects, we placed two small 10 × 10 × 20 cm^3^ (L × W × H) objects in a 100 × 100 cm^2^ arena (Fig. 4). We found that the boundaries of the objects also elicited strong responses in mPFC border cells (Fig. 4 *A*–*C*), with a mean firing rate around the objects comparable to that along the walls (Fig. 4 *E* and *F*, *n* = 9, two-sided Wilcoxon signed rank test, 2.21 ± 0.9 Hz versus 2.24 ± 1.01 Hz, Z = 0.14 and *P* = 0.89). Moreover, the CW/CCW ratio in firing rate around the objects was reversed compared to that along the geometric borders. For left egocentric border cells, the CW/CCW ratio in firing rate around the objects was lower than 0.5, while for right egocentric border cells, the value was larger than 2, opposite to those around the four walls of the environment (Fig. 4*G*). This was also reflected in the preferred directions along the four borders of the square arena and the small objects (Fig. 4*D*), further confirming the egocentric nature of spatial coding. This observation was consistent with the responses of annulus cells from the ACC that fire preferentially around the environment’s periphery (32), which often exhibited an increased firing rate around objects placed in the arena. Altogether, these results suggest that mPFC border cells encode the general geometric borders of the external environments.

### Stable Tuning of mPFC Border Cells in Darkness.

**Fig. 5.**
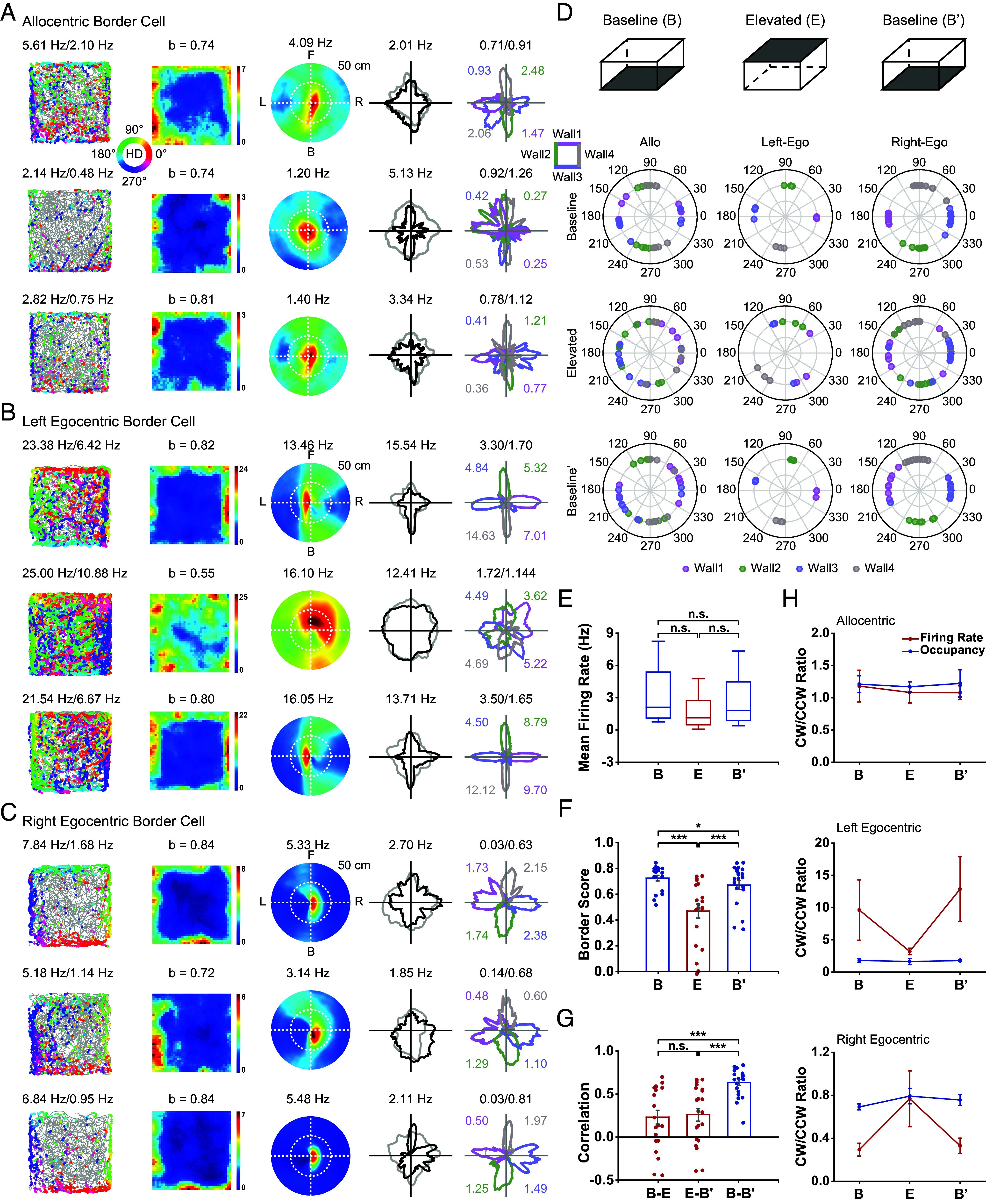
mPFC border cells persist in darkness. (*A*–*C*) Spatial responses of representative allocentric (*A*), left egocentric (*B*), and right egocentric (*C*) border cells, respectively, in the light (L), dark (D), and light (L’) conditions. Notations and symbols are similar to those in Fig. 2. (*D*) *Top*, diagram of experimental procedure with the light, darkness, and light conditions. *Bottom*, polar plot showing the distribution of peak direction of head-direction tuning along color-coded walls under those three conditions. (*E* and *F*) Both mean firing rate and border score were not significantly different between darkness and light conditions. (*G*) The spatial correlation of firing rate maps remained high between darkness and light conditions. (*H*) The CW/CCW firing rate ratio remained almost unchanged in total darkness compared to the light condition. Curves in blue represent time_cw/ccw and curves in red represent fr_cw/ccw.

The mPFC is known to interact with sensory regions and exhibit sensory-evoked responses to visual stimuli (44, 45). To determine whether visual input was essential for maintaining spatial tuning of mPFC neurons, we performed sequential recordings from 19 border cells in the dim light (L), darkness (D), and dim light (L’) conditions (Fig. 5). Darkness did not affect on the mean firing rate (Fig. 5, two-sided Wilcoxon signed rank test, L-D, Z = −0.36 and *P* = 0.72; D-L’, Z = −1.09 and *P* = 0.28; L-L’, Z = −1.09 and *P* = 0.33) or border score (Fig. 5, same test, L-D, Z = −1.61 and *P* = 0.11; D-L’, Z = −0.32 and *P* = 0.75; L-L’, Z = −1.76 and *P* = 0.08). The spatial correlation of firing rate maps between light and dark conditions showed no significant change (Fig. 5*G*, same test, L-D, Z = −0.89 and *P* = 0.38; D-L’, Z = −1.49 and *P* = 0.14; L-L’, Z = −0.40 and *P* = 0.69).

Specifically, the directional tuning of both left and right egocentric border cells (*n* = 4 and 10, respectively) was retained with the preferred direction along four walls remaining nearly unchanged in the absence of visual input (Fig. 5*D*), and the CW/CCW ratio in firing rate also remained stable under darkness (Fig. 5*H*). Overall, these results suggested that both allocentric and egocentric tuning properties of mPFC border cells are not dependent on visual sensory input.

To further test whether mPFC border cells were induced by tactile responses, we trimmed the rat’s whiskers and recorded border responses (*SI Appendix*, Fig. S10). No significant change in the mean firing rate or in the border score before, after one-side whisker trimming, and after two-side whisker trimming was observed. These results suggest that whisker sensation is not essential for mPFC border representations.

### Dependence of mPFC Border Responses on Physical Boundary.

**Fig. 6.**
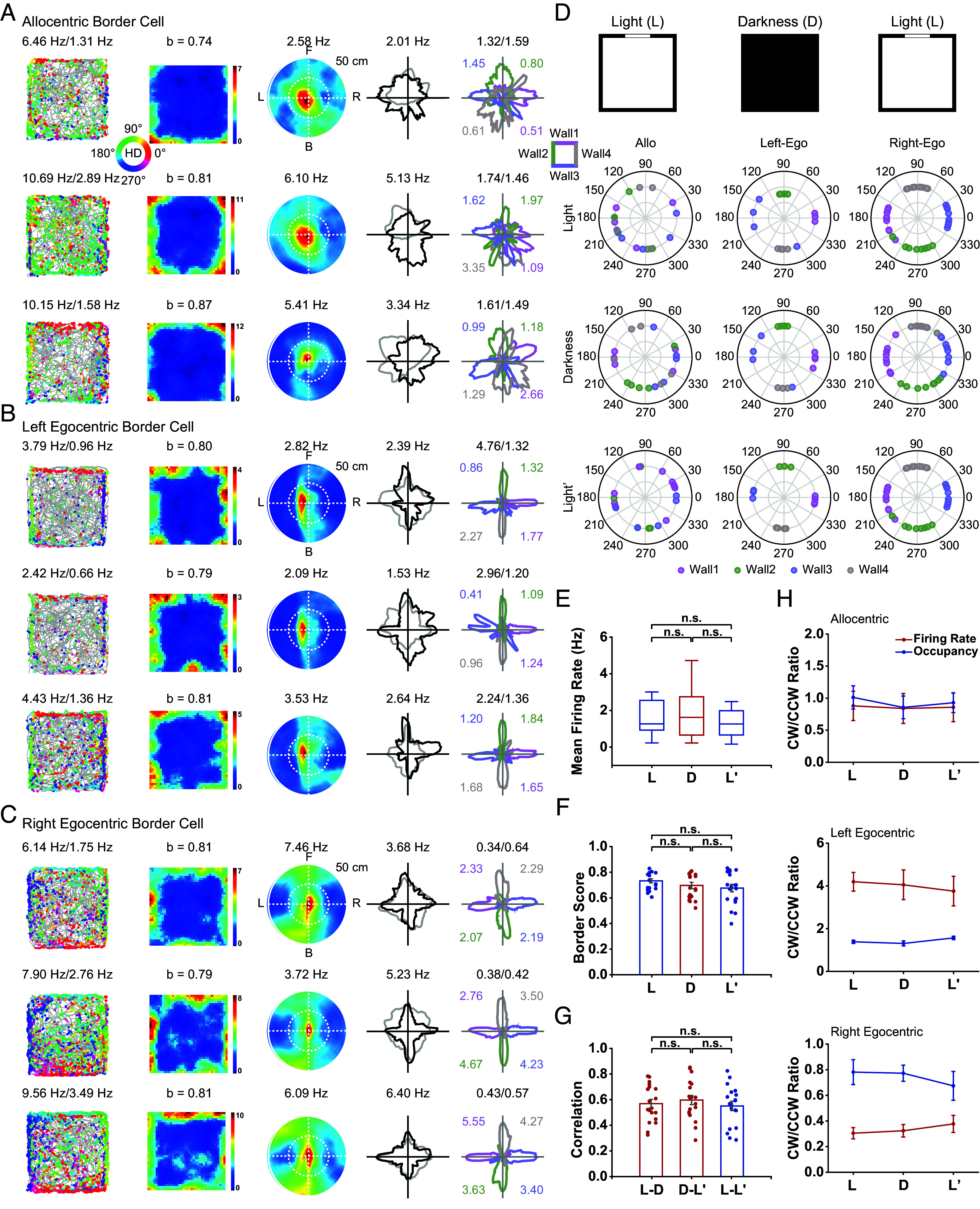
mPFC border cells require geometric physical borders. (*A*–*C*) Spatial responses of representative allocentric (*A*), left egocentric (*B*), and right egocentric (*C*) border cells, respectively, in the baseline (B), elevated platform without walls (E), and baseline (B’) sessions. Notations and symbols are similar to those in Fig. 2. (*D*) *Top*, diagram of experimental procedure with the baseline, elevated, and baseline conditions. *Bottom*, polar plot showing the distribution of peak direction of head-direction tuning along color-coded walls under those three conditions. (*E* and *F*) Comparison of mean firing rates (*E*) and border score (*F*) of border cells between baseline and elevated platform. Border score was significantly reduced in elevated wall-less platforms. (*G*) Spatial correlation of firing rate maps between baseline and elevated conditions decreased significantly. (*H*) mPFC border cells didn’t maintain CW/CCW firing rate ratio in environments without physical borders. Curves in blue represent time_cw/ccw and curves in red represent fr_cw/ccw.

We investigated whether mPFC border cells also respond to boundaries other than physical walls by using an elevated platform with a 50-cm drop on four sides. Similar to their counterparts in MEC or subiculum (13, 17), the mPFC border could still be identified along drop edges (Fig. 6 *A*–*C*), suggesting that mPFC border cells respond to general environmental boundaries. Specifically, 8 out of 21 cells preserved their border tuning in the elevated platform, indicating that mPFC border cells were not simply driven by somatosensory signals. Although the mean firing rate did not change significantly in the drop-edge platform (Fig. 6*E*, *n* = 21, two-sided Wilcoxon signed rank test, B-E, Z = −1.13 and *P* = 0.26; E-B’, Z = −0.71 and *P* = 0.48; B-B’, Z = −0.44 and *P* = 0.66), their border score decreased (Fig. 6*F*, same test, B-E, Z = −3.60 and *P* < 0.001; E-B’, Z = −3.16 and *P* < 0.002; B-B’, Z = −2.42 and *P* = 0.02). This was accompanied by a sharp drop in spatial correlations between firing rate maps of baseline session versus elevated platform session, while correlations remained high between two baseline sessions (Fig. 6*G*, same test, B-E versus E-B’, Z = −1.10 and *P* = 0.27; E-B’ versus B-B’, Z = −3.77 and *P* < 0.001; B-E versus B-B’, Z = −3.77 and *P* < 0.001). A previous study reported a type of subicular boundary vector cells that responded to drop edges similarly to walls (46), which differed from the observation here that mPFC border cells relied on physical walls.

Notably, the directional tuning of both left and right egocentric border cells (*n* = 3 and 10, respectively) was disrupted in the elevated platform, with the preferred direction along four walls not following a 90° step change as in the baseline condition (Fig. 6*D*). Meanwhile, the CW/CCW ratio in firing rate was also interrupted (Fig. 6*H*). Together, these results suggested that the spatial tuning of mPFC border cells relies on physical boundaries, similar to previously reported border response properties in MEC (13, 17).

### Persistence of mPFC Border Responses across Different Environments.

Distinct spatial cells like place cells and grid cells, vary their firing patterns across different environments (47, 48). To determine whether spatial responses of mPFC border cells depend on environmental context, we compared the activity of 15 border cells in square recording boxes in separate familiar and novel rooms (*SI Appendix*, Fig. S11). Similar to border cells in other brain regions (13, 17), mPFC border cells were insensitive to context, with no significant change between the novel and familiar environments in their mean firing rate, or border score. Moreover, the alteration of geometric shape from square to circle did not change the mean firing rate of mPFC border cells, but resulted in a higher border score in the circular arena than that in the square enclosure (*SI Appendix*, Fig. S12), likely due to the uniform boundary of circular environment. Jointly, these results demonstrated that mPFC border cells are insensitive to environmental geometry and novel circumstances.

### Near Absence of Theta-Rhythmicity of mPFC Border Cells.

Theta oscillations (4 to 12 Hz) are a prominent feature of neural activity observed in the mPFC during spatial working memory-related tasks, both in the local field potential and in the spiking of single neurons (49, 50). Theta coherence is observed in hippocampal-prefrontal networks during memory-guided spatial tasks (23, 51–57), while hippocampal theta rhythm is thought to be critical for spatial computations and temporally codes spatial location by the precession of spike timing relative to theta oscillations termed theta phase precession (58–60). However, it has yet to be determined whether theta rhythmic modulation of mPFC border cells exists during freely foraging behavior.

**Fig. 7.**
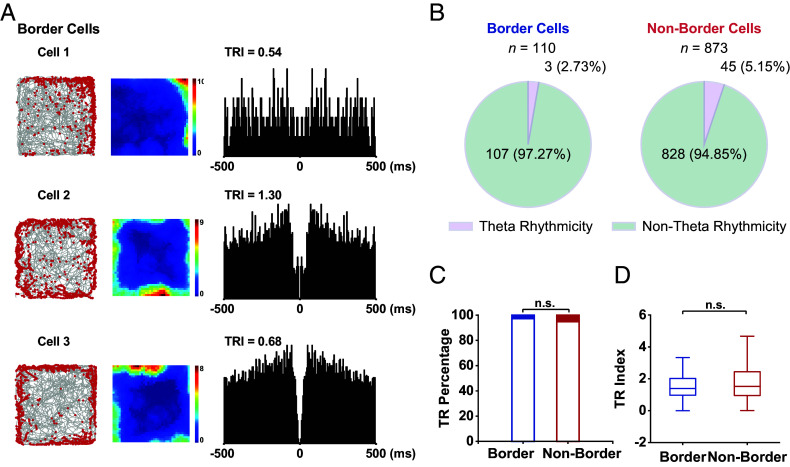
mPFC border cells show little theta rhythmicity. (*A*) Three representative mPFC border cells from Fig. 1*B* and their spike-train autocorrelograms. *Left*, trajectory (gray line) with superimposed spike locations (red dots); *Middle*, firing rate maps; *Right*, spike-train autocorrelograms with TRI labeled at the *Top* of each panel. (*B*) Pie diagrams showing the proportion of theta-rhythmic cells for border and nonborder cells. (*C*) The fraction of border cells showing theta rhythmicity was similar to that of nonborder cells. Filled portions of the bars represent the proportions of theta-rhythmic cells and unfilled portions represent non-theta-rhythmic cells. (*D*) The TRI for the border versus nonborder cells was comparable.

To investigate whether the neuronal activity of mPFC border cells was theta modulated, we computed theta rhythmicity index (TRI) and quantified the difference in theta rhythmicity between border and nonborder cells. mPFC cells rarely showed theta rhythmic firing during freely forging behavior (Fig. 7*A*). Altogether, only 3 out of 110 (2.73%) of border cells exhibited clear theta rhythmicity, while 5.15% (45/873) of nonborder cells showed theta rhythmicity (Fig. 7*B*). The ratio of theta-rhythmic border cells was comparable to that of nonborder cells (Fig. 7*C*, chi-squared test, *P* = 0.27). Moreover, the mean TRI between mPFC border cells and nonborder cells was also not statically different (Fig. 7*D*, two-sided Mann–Whitney *U* test, *n* = 110 versus 873, Z = −1.04, *P* = 0.30). The near absence of theta rhythmicity of mPFC border cells indicated that geometric border representation may be independent of theta-entrainment from the hippocampus.

## Discussion

Here, we demonstrated that the ventral part of the mPFC, composed of prelimbic and infralimbic cortices, displayed geometrical border tuning during freely foraging behavior. Together with previous reports of spatially tuned neurons in visual (61–65), somatosensory/sensorimotor (63, 66), olfactory (67), orbitofrontal (68), retrosplenial cortex (69–71), claustrum (72), posterior parietal (73–76), postrhinal cortex (77, 78), and other sensory and higher-order brain areas (79, 80), our findings further contribute to the notion that spatial tuning might be a broad feature of cortical computation (81). Moreover, mPFC border cells add to the boundary or border responses already reported in MEC (13, 18, 82), subiculum (16, 17, 46, 83), anterior claustrum, and nucleus reuniens (72).

The mPFC is responsible for many aspects of higher cognitive functions such as working memory, decision-making, goal-oriented behaviors, and attentional selection of task-relevant information (1, 22). These functions critically require the instantaneous spatial information of one’s own location and surrounding environment (84). Geometric boundaries might serve as references for path-integration-based position estimates, with resetting of the path integrator and subsequent reduction of error taking place regularly near major boundaries or landmarks (85–87). Thus, mPFC border cells could aid in the process of planning guide for selecting a navigational route (11). Previous studies have revealed mPFC to encode the spatial location of goals or rewards in a task rule-dependent manner (23–27), a possible reflection of task-phase coding (88). Spatially tuned activities were also examined in freely moving animals (27, 29). Earlier recordings investigated the responses of 31 cells from the medial precentral area (PrCm), dorsal anterior cingulate cortex (ACd), and prelimbic and infralimbic area, and found no place-specific firing characteristics reminiscent of hippocampal place cells in the random food search task in the square chamber (27). Another study identified 18 units from prelimbic areas with low firing rates ranging from 0.03 to 1.31 Hz, showing no spatially related firing (29). Despite that, recording from the prelimbic region (28) and the ACC (32) revealed cells firing specifically around the periphery or the center of the environment. This discrepancy among previous studies might be due to the relatively limited sample size or lack of border-firing investigation (27, 29). A recent study reported spatial tuning of locations emerged in rodent mPFC during virtual navigation on a circular path (34), which supports mPFC spatial tuning without engaging of particular task demand.

Regarding the discrepancy between previous studies and the current study, several factors may contribute to this. First, the current study mainly targeted ventral mPFC, including the prelimbic and infralimbic regions. Tracing studies have shown that the infralimbic region of mPFC receives more projections from the hippocampus (8, 9, 89, 90), while functional studies have shown that the dorsal region exhibited higher spatial tuning (34) and greater context differentiation (91). Whether the dorsal part of mPFC, including the ACC, also encodes spatial information under naturally exploring states remains to be determined although a previous study has reported that ACC neurons fired preferentially in the center or around the periphery of the arena, termed bulls-eye and annulus cells, respectively (32). Second, mPFC processes long-range inputs from many other brain regions, including the thalamus, basolateral amygdala (BLA), ventral hippocampus (vHPC), and claustrum in a layer-specific targeting manner (92), which may contribute to a layer-dependent manner of mPFC border representation. For instance, projection from vHPC and MEC mainly target layer V, while axons from BLA preferentially target layer II. Since we mainly recorded from the deep layer of mPFC, further investigations of the superficial layer II/III of the mPFC will help elucidate whether mPFC border cells exhibit a layer-dependent distribution. Third, the restrained head movement or specific setup of running tracks in previous studies might compromise the spatial tuning properties observed in open arenas under the current study, which put little restriction on animal behavior.

The interplay between the hippocampus and mPFC is widely recognized to play a central role in various behavioral and cognitive functions (6). The mPFC receives direct input from CA1 (90), and direct hippocampal–prefrontal afferents are known to be critical for mPFC neurons to encode spatial cues (93). This pathway may also contribute to the topographically organized place code observed in mPFC (34). Increasing evidence has suggested theta rhythms may facilitate hippocampal outputs to the mPFC during spatial working memory tasks (49, 54, 55, 94) and mPFC theta power was selectively enhanced in a context-dependent manner. An increase in theta oscillations in mPFC and hippocampal–prefrontal LFP synchronization was observed only during the exploration of the incongruent object (95). Even during the choice phase in spatial working memory tasks, theta power in mPFC was much less significant than that in the hippocampus (96). We found nearly absent theta rhythmicity of mPFC border cells and nonborder cells during spontaneous locomotion, in consistent with weak theta entrainment from the hippocampus under task- or rule-free conditions. Further investigation using optogenetic manipulation of the hippocampal-prefrontal pathway will shed light on the influence of hippocampal formation on the spatial tuning of mPFC neurons.

Another intriguing finding of the current study is the coexistence of allocentric and egocentric spatial representations within mPFC. Sensory information about the surrounding environment enters the brain via sensory organs in egocentric coordinates relative to the animal itself. This information is then incorporated into a stored spatial map in allocentric coordinates (97). In turn, when stored allocentric representations are invoked to guide navigation, it would require translation into sequences of actions anchored in an egocentric reference frame (98). Computational models have predicted that neurons with egocentric sensitivity to external locations would enable the integration of allocentric and egocentric information for either constructing or using stored spatial representations (99, 100). Thus, these dual encodings of two reference frames within mPFC might support local spatial computation for coordinate transformation to guide context-dependent behavior. The clear lateralization of mPFC egocentric border tuning contralateral with the hemisphere that the neuron is within reflects the nature of sensory origin, as mPFC receives multimodal cortico-cortical projections from the motor, somatosensory, visual, auditory, gustatory, and limbic cortices (101). In conclusion, our results reveal the coexistence of allocentric and egocentric boundary tuning in mPFC, supporting the local circuit computation for the execution of spatial memory–related tasks.

## Materials and Methods

### Implanted Rats.

Ten male Long-Evans adult rats were implanted with a self-assembled microdrive loaded with four tetrodes to target the prelimbic and infralimbic cortices of mPFC region (∼0.5 to 1 mm lateral to the midline, ∼3 to 5 mm anterior–posterior from bregma, ∼1.5 to 2.0 mm dorsal–ventral below the dura and at an angle of 5 to 10° from the medial-to-lateral direction in the coronal plane).

### Behavioral Protocol and Data Collection.

Rats were trained to forage in different shapes of enclosures with a white cue card (297 × 210 mm^2^) mounted on one side of the interior wall. Data were acquired by an Axona system (Axona Ltd., St. Albans, U.K.). Details of environment manipulation, spike sorting, construction of the spatial firing rate map and calculation of theta rhythmicity are described in *SI Appendix*.

### Identification of mPFC Border Cells.

The calculation of the border score was followed by previous publications (13, 16, 102, 103). Details of quantification of border and directional tuning are described in *SI Appendix*.

### Quantification of Egocentric Tuning.

EBRs were constructed using previously described methods (42, 43). The mathematics for calculating egocentric tuning strength is described in *SI Appendix*.

### Histology and Tetrode Track Location.

After the final recording session, the rat’s brain was sectioned and stained with Cresyl Violet for the visualization of the final recording positions according to the sixth edition of *The Rat Brain in Stereotaxic Coordinates* (104). The detailed procedure for Nissl-staining is summarized in *SI Appendix*.

## Supplementary Material

Appendix 01 (PDF)

## Data Availability

All codes and raw data utilized for analysis in the article and/or *SI Appendix* are available on GitHub (105) and can be found at this link: https://github.com/Zhang-Sheng-Jia-Lab/mPFC_Code.
