# Supplementary material for: Border cells without theta rhythmicity in the medial prefrontal cortex
Source: Proc Natl Acad Sci U S A. 2024 Jun 10;121(25):e2321614121. doi: 10.1073/pnas.2321614121 (PMC11194599; doi:10.1073/pnas.2321614121)
Supplement: Supplementary file 1 — Appendix 01 (PDF) [file pnas.2321614121.sapp.pdf]

Supplementary Information for

Border cells without theta rhythmicity in the medial prefrontal cortex

Xiaoyang Long[a,1], Bin Deng[a,1], Rui Shen[a,1], Lin Yang[a,1], Liping Chen[a,1], Qingxia Ran[a], Xin Du[a], and Sheng-Jia Zhang[a,2]

Author Affiliations:

[a]Department of Neurosurgery, Xinqiao Hospital, Army Medical University, Chongqing 400037, China

[1]These authors contributed equally to this work.

[2]To whom correspondence may be addressed. Email: sheng-jia.zhang@outlook.com or sheng-jia.zhang@tmmu.edu.cn (S.-J.Z.)

This PDF files includes:

Table S1

Supplementary Figs. S1 to S12

Supplementary References

Supplementary Materials and Methods

Materials and methods in this investigation were similar to those described previously (1-3).

Subjects

Ten male Long-Evans adult rats (aged 2-4 months, weighing 250-450 grams on the day for chronic surgery) were implanted for chronic recording experiments. Rats were housed individually in transparent plexiglass cages (W × L × H: 35 cm × 45 cm × 45 cm) and kept on a reversed 12-hour light/12-hour dark schedule (lights on from 21:00 p.m. to 09:00 a.m. hours). All recording trials were performed during the dark phase. Rats were kept in a temperature-controlled (19-23°C) and humidity-adjusted (55-70%) vivarium and rats were placed on a partially food-deprived schedule to about 85-90% of free-feeding body weight. Food restriction was imposed 8-24 hours before each training and recording trial. Water was supplied *ad libitum*. The animal experiments were approved by the National Animal Welfare Act under a protocol with the permission license number #SYXK-2017002 in accordance with the Animal Care and Use Committee from the Army Medical University and Xinqiao Hospital.

Surgery and Electrode Placement

Tetrodes were constructed with four twisted 17 µm polyimide-coated platinum-iridium (90-10%) wires (#100167, California Fine Wire Company, USA). Tetrodes were plated with a 1.5% platinum solution to reduce electrode impedances to between 150 and 300 kΩ at 1 kHz through electroplating before surgery (nanoZ; White Matter LLC, Seattle, Washington, USA). Anesthesia was induced by isoflurane mixed with oxygen (1.5-3.0% in O_2), immobilized in a stereotaxic frame (David Kopf Instruments, Tujunga, California, USA) and kept on a feedback-adjusted temperature control pad at 37°C to maintain stable body temperature. A self-assembled microdrive loaded with four tetrodes was

implanted to target the prelimbic and infralimbic cortices of mPFC region (~0.5-1 mm lateral to the midline, ~3-5 mm anterior-posterior from bregma, ~1.5-2.0 mm dorsal-ventral below the dura and at an angle of 5-10° from the medial-to-lateral direction in the coronal plane). Dental cement together with 8-10 anchor screws was used to secure the microdrive to the rat brain surface with one screw behind the eyes connected to the system ground serving as the ground electrode.

Behavioral Protocol and Data Collection

Both unit activity and local field potentials (LFP) were recorded in parallel. The pre-training started about one week after the recovery during which one or two sessions were recorded each day for another week before the experiment started. Rats were trained to forage in different shapes of enclosures with a white cue card (297 × 210 mm^2) mounted on one side of the interior wall. Food pellets were randomly dispersed into the enclosure intermittently to encourage free exploration. To avoid large electrical noise due to chewing, the food pellets (vanilla-flavored biscuits) were crumbled into small pieces before being thrown randomly into the running arena. Usually, ~3-6 g food pellets were used during one recording session. To calculate the heat maps of distribution of food pellets, the images after random food distribution procedure without the rats in the running arena was subtracted from the images taken before that and transformed to an 8-bit grayscale images. Then, the arena was divided into two areas with the same size: the inner and outer zone. The average grayscale values in these two zones were calculated, normalized by 256 and compared.

In the open field, each recording trial lasted typically between 20 and 40 min to facilitate full coverage of the testing enclosure. Tetrodes were lowered very slowly in steps of 25 or 50 μm until well-separated single units could be identified. Data were acquired by an Axona system (Axona Ltd., St. Albans, U.K.) at 48 kHz, band-pass filtered between 0.8-6.7 kHz and a gain of 5,000-25,000 times. Spikes were digitized with 50 8-bit sample windows. Local field

potentials were recorded from one of the electrodes with a low-pass filter at 500 Hz.

Spike Sorting, Cell-Type Identification and Firing Rate Map

To identify well-isolated units, spike sorting was manually performed offline with graphical cluster-cutting software (TINT, version 4.4.16, Axona Ltd, St. Albans, U.K.), and the clustering was primarily based on features of the spike waveform (peak-to-trough amplitude and spike width), together with additional autocorrelations and cross-correlations separation tools and criteria. During the manual cluster cutting, units with similar or identical waveform shapes were strictly counted only once whenever similar or identical individual units were recorded and tracked across two consecutive recording trials. Only recording trials in which rats covered more than 80% of the running enclosure were taken for further analysis. Putative fast-spiking interneurons were classified on the basis of spike waveform and firing rate (4, 5). A mixture of two Gaussians was fitted to the distribution and the local minimum was used as the cutoff for the waveform classification (6, 7).

A pair of small light-emitting diodes (LEDs) were attached to the headstage to track the rats' speed, position and head orientation via an overhead video camera. Only spikes recorded during the animal's instantaneous running speeds > 2.5 cm/s were chosen for further analysis to exclude confounding behaviors such as immobility, grooming or rearing.

To classify firing fields and firing rate distributions, the position data were divided into 2.5 $\times$ 2.5 cm^2 bins, and the path was smoothed with a 21-sample boxcar window filter (400 ms; 10 samples on each side). Units with > 100 spikes per session and with a coverage of >80% were included for further analyses. Maps for spike numbers and spike times were smoothed with a quasi-Gaussian kernel over the neighboring 5 bins $\times$ 5 bins. Spatial firing rates were calculated by dividing the smoothed map of spike numbers by spike times. The peak firing

rate was defined as the highest rate in the corresponding bin within the spatial firing rate map. Mean firing rates were averaged from the whole session data. The smoothed rate maps were plotted as a color-coded image using 256 colors to scale with. The color in each spatial bin was determined by the firing rate normalized to the peak firing rate. The spatial stability was estimated by computing spatial correlations between two smoothed rate maps for the first and second halves of the same recording trial.

Identification of mPFC Border Cells

The calculation of the border score was followed by previous publications (3, 8-10). Briefly, putative firing fields were identified by identifying any continuous area with neighboring pixels whose firing rates are higher than 0.3 times the maximum firing rate and cove a total area of at least 200 cm^2. Then, the maximum coverage of any field with pixels along a particular wall was determined. Meanwhile, the average distance over all pixels from this firing field to the nearest wall was quantified, weighted by the firing rate and normalized by the sum. Finally, border cells were identified by calculating the border score, or the difference between the maximal length of any of the four walls touching on any single spatial firing field of the cell and the average distance of the firing field to the nearest wall, divided by the sum of those two values. The border score ranged from −1 for cells with perfect central firing fields to +1 for cells with firing fields that exactly line up with at least one entire wall.

Border cell classification was verified using population shuffling methods. For each permutation trial, the whole sequence of spike trains was time-shifted along the animal’s trajectory by a random period between 20 s and 20 s less than the length of the entire trial, with the end wrapped to the start of the trial. A spatial firing rate map was then obtained, followed by border score estimation.

The distribution of border score was calculated for the entire set of permutation trials from all recorded units, from which the threshold with the 99th percentile

was determined. The unit was defined as a border cell if its border score was higher than the 99th percentile threshold derived from the shuffled data.

Quantification of Head-Direction Tuning

The rat's head direction was computed by the relative position of a pair of LEDs differentiated through their sizes. The directional tuning curve for each recorded unit was drawn by plotting the firing rate as a function of the rat's head angle, which is divided into bins of 3° and then smoothed with a 15° mean window filter (2 bins on each side). The strength of directionality was measured by computing the mean vector length from the circularly distributed firing rates. The calculation of mean vector length (mvl) was followed by previous publications (6, 8, 10, 11). Head direction tuning along four walls of border cells was measured in a 15-cm-wide area along each wall.

$$\mathrm{MV} = \frac{\sum_i^n F_i * e^{i*\theta_i}}{\sum_i^n F_i}$$

where F_i was the firing rate in the bin *i*, θ_i was the head direction angle in the bin *i*, *n* is the total number of directional bins. The mean vector length is defined as the absolute value of MV (mean vector) and is used to quantify the strength of head direction tuning. The preferred angle of directional tuning was then determined as the head direction angle with the highest firing rate across all directional bins. While head direction was derived from the angle between two tracking LEDs of different sizes mounted on the animal's head, movement direction was calculated as the instantaneous derivative of the tracked position.

Quantification of Egocentric Tuning

To test the egocentric tuning property of border cells, we constructed egocentric boundary ratemaps (EBRs) following previously described methods (12, 13), which illustrate the orientation and distance of the geometric borders relative to the animal's head direction when a given cell fires. Briefly, for each video frame,

the intersection from the animals’ current location to the nearest geometric borders from the animal’s current direction in steps of 3° over 360° was calculated. The head direction or movement direction was used as the reference for the angular bins. The distance was binned at 2.5 cm and the distances were only considered if their length was smaller than half the size of the running arena. The procedure was repeated for each spike emitted by a given cell and each time sample the animal has occupied, and by dividing the number of spikes by the amount of occupancy, an egocentric boundary ratemap in polar coordinates was constructed.

To calculate the strength of egocentric tuning, we computed the mean vector as

$$\mathrm{MV} = \frac{\sum_{\theta}^{n} \sum_{d}^{m} F_{\theta,d} * e^{i*\theta}}{\sum_{\theta}^{n} \sum_{d}^{m} F_{\theta,d}}$$

Where θ is the angular bin relative to the animal’s head direction or movement direction, d is the distance bin from the animal’s position, $F_{\theta,d}$ is the firing rate in the θ-d bin, n is the total number of orientation bins, m is the total number of distance bins. The mean vector length is defined as the absolute value of MV and is used to quantify the strength of egocentric tuning. The preferred angle of egocentric tuning was then determined as

$$\text{Preferred Angle} = \mathrm{arctan2}\left(\frac{\mathrm{Imaginary(MV)}}{\mathrm{Real(MV)}}\right)$$

Environmental Manipulations

For recording in the elevated platform without walls, we first recorded mPFC units in the square box, followed by the recording in the elevated platform without walls. For recording in the dark, we first recorded mPFC units in the square box under the light condition, followed by the recording in the dark and back to the light condition. For recording in different geometric shapes, we first recorded mPFC units in the square box, followed by the recording in the circular

enclosure and back to the square box. For the recording of border cells in the presence of an inserted wall, a baseline recording trial was performed followed by the additional introduction of one discrete wall in three different orientations: from horizontal to vertical to diagonal, then ended with another baseline recording session. For the object session, one or two objects (10 × 10 × 20 cm^3) were placed in the running arena.

The inter-session interval was approximately 10 minutes for cleaning the running area and manipulating the environments while the rat was not present in the recording box but kept in a holding enclosure. To remove any possible olfactory cues, we cleaned the running environment with an alcohol solution and water before each session. To reduce the possible influences of the surrounding environment, we first disorientated the rats and then placed them on the running box floor in a random direction.

Theta Rhythmicity

The local field potentials (LFPs) was filtered to extract theta oscillations. For the low-pass filtering, 4 and 5 Hz were selected as stopband and passband low cut-off frequencies, respectively, while 10 and 11 Hz were selected as passband and stopband high cut-off frequencies, respectively. Theta rhythmicity was calculated from the fast Fourier transform (FFT)-based power spectrum of the spike-train autocorrelation. When the mean spectral power within 1 Hz range of the theta peak within the 4-11 Hz frequency range was at least 5 times larger than the mean spectral power from 0 Hz to 125 Hz (the ratio defined as theta rhythmicity index, TRI), the cell was classified as being theta rhythmic.

Histology and Tetrode Track Location

After the final recording session, rats were deeply anesthetized with an overdose of sodium pentobarbital and perfused intracardially with ice-cold 1 × phosphate-buffered saline (PBS) followed by a 4% ice-cold paraformaldehyde (PFA) in 1 × PBS solution. Afterwards, the brains were taken out and post-fixed

in a 4% PFA in 1 × PBS solution at 4°C for more than 24 hours. The electrolytic lesions of five brains (SI Appendix, **Figs. 1E-I**) were made by passing 20-30 μA current for 15-20 seconds through two of the channels on the electrodes before the perfusion. The brain was then transferred into 10, 20 and 30% sucrose/PFA solution sequentially across 72 hours before sectioning by using a cryostat. Thirty-micron-thick coronal sections were serially cut and obtained through the targeted brain area. Sections were mounted on glass slides and stained with Cresyl Violet (Sigma-Aldrich). The final recording positions were imaged and determined from digitized images of the Nissl-stained sections scanned with the Olympus Slideview VS200 Digital Slide Scanner. Positions of each recording were estimated from the deepest tetrode track according to the daily notebook on tetrode advancement. The tissue shrinkage correction was calculated by dividing the distance between the brain surface and electrode tips by the final advanced depth of the recording electrodes. Electrode traces were confirmed to be located in mPFC based on the reference figures published in the sixth edition of *The Rat Brain in Stereotaxic Coordinates* (14).

Data, Materials, and Software Availability: All source codes and raw data utilized for analysis in the article and/or SI Appendix are available on GitHub and can be found at this link: https://github.com/Zhang-Sheng-Jia-Lab/mPFC_Code.

Table S1. Summary of the recorded cell number and brain hemisphere in each animal.

Animal#	Border cells	Total cells	Hemisphere
Rat# A	7	32	Right
Rat# B	2	38	Left
Rat# C	7	74	Right
Rat# D	20	133	Left
Rat# E	40	202	Left
Rat# F	2	9	Right
Rat# G	2	61	Left
Rat# H	3	83	Right
Rat# I	12	96	Left
Rat# J	15	255	Left

*Rat# A-# I correspond to the animals shown in **Supplementary Figs. S1A-I**. Rat# J corresponds to the rat shown in **Fig. 1A**.

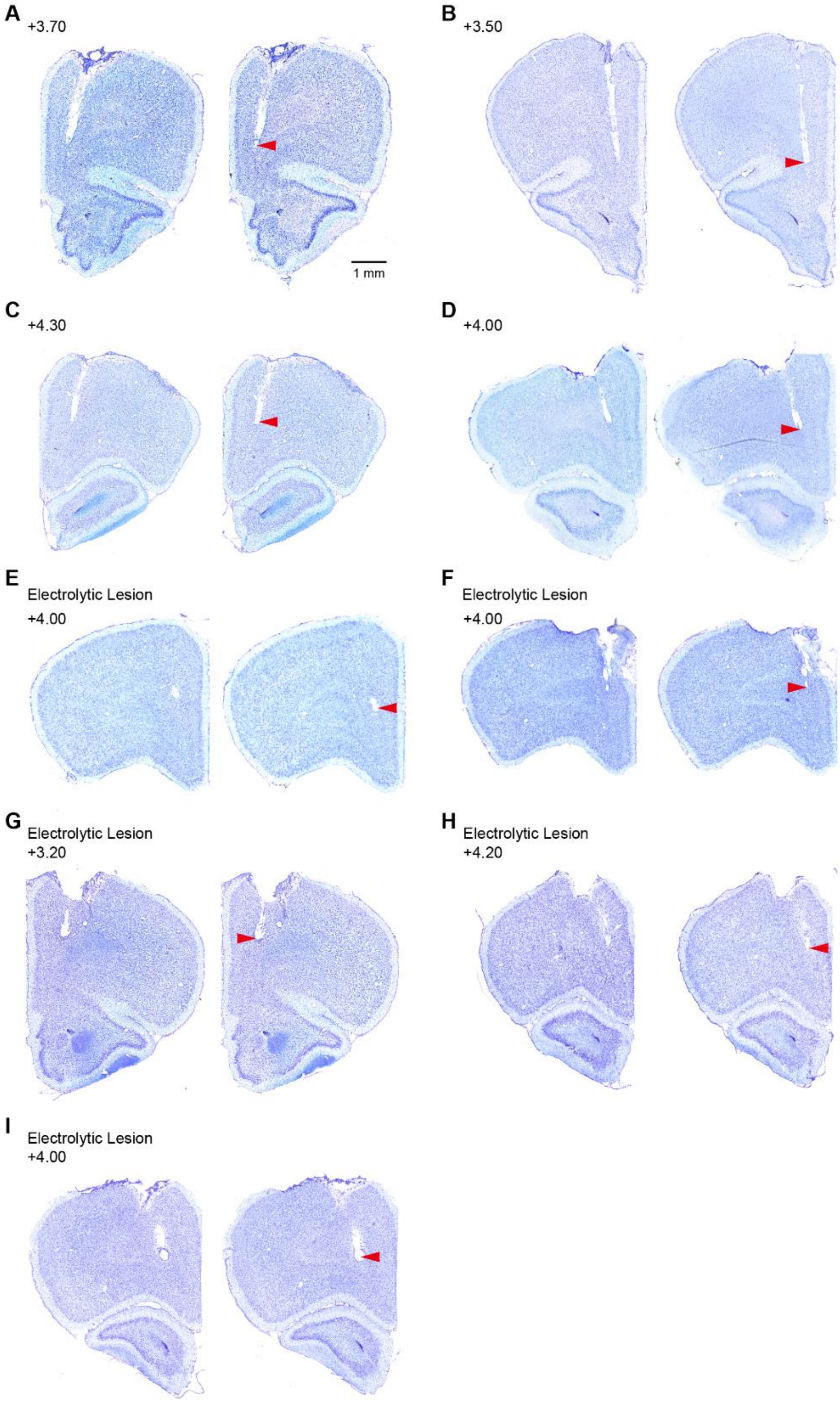

Supplementary Fig. S1. Nissl staining confirmed the location of the electrode tip in mPFC.

(**A-I**) Nissl-staining results from an additional nine rats showing the final recording location of the electrode tips in mPFC from two consecutive coronal brain sections. Red arrows indicate the deepest location of the electrode track. Scale bar, 1 mm. Additional electrolytic lesions were performed in five brains shown in **E-I**.

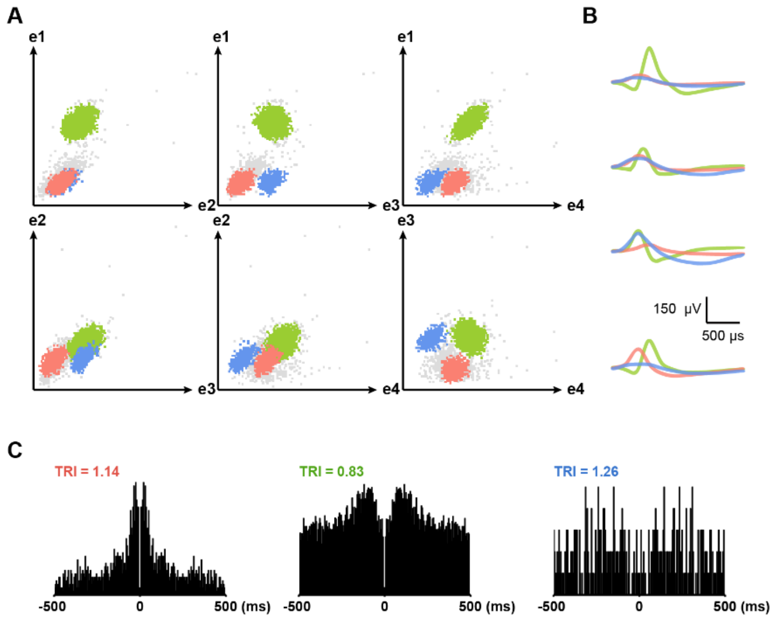

Supplementary Fig. S2. Cluster separation.

(**A**) Scatterplots from one representative recording session showing the relationship between peak-to-trough amplitudes of all spikes on six combinations of channels from four tethered electrodes (e1-e4) on a specific tetrode. Each dot represents a single spike. Each color indicates one well-separated cluster, while the grey dots indicate unclustered spikes. (**B**) Waveforms on four electrodes from the three separated clusters in (**A**). (**C**) The spike-train autocorrelograms for the three representative cells in (**A**) with theta rhythmic index (TRI) labeled at the top left of the panel.

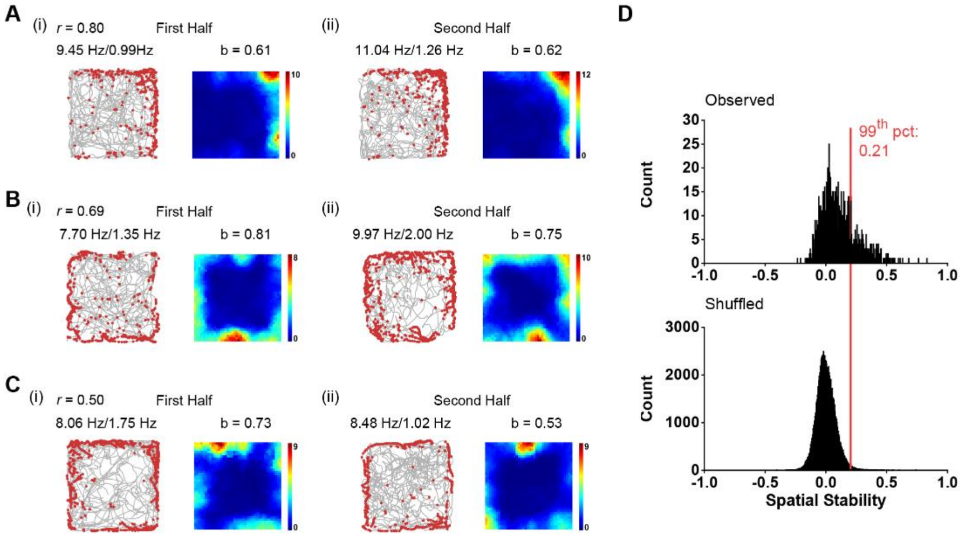

Supplementary Fig. S3. Spatial stability of mPFC border cells.

(**A-C**) Spatial responses for the first half and second half of the same recording sessions for three border cells in **Fig. 1B**. Correlation coefficient (*r*) was labeled at the top of the panels. (**D**) Distribution of spatial stability for observed data (top panel) and shuffled data (bottom panel). The red line indicates the 99th percentile for spatial stability derived from the shuffled data.

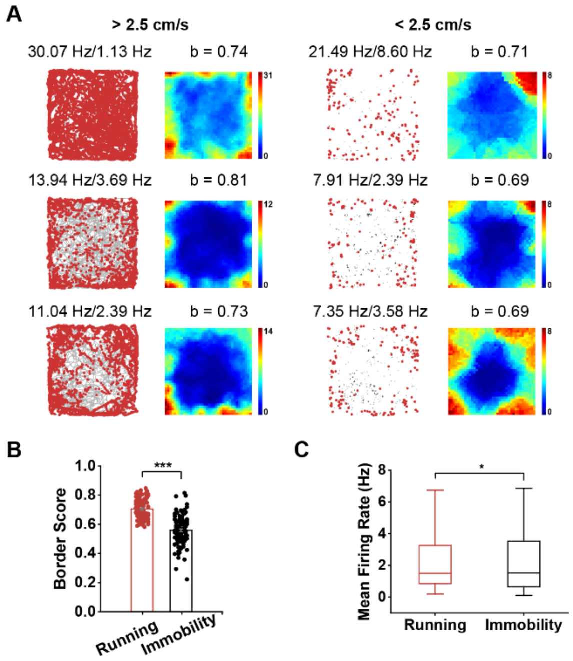

Supplementary Fig. S4. Spatial responses of mPFC border cells during running and immobility.

(**A**) Spatial responses for the first half and second half of the same recording sessions for three border cells. (**B**) Comparison of border score during running and immobility (n = 110, two-sided Wilcoxon signed rank test, 0.70 ± 0.01 versus 0.56 ± 0.01, Z = -8.65 and ***p < 0.001). (**C**) Comparison of mean firing rate during running and immobility (same test, 2.74 ± 0.27 Hz versus 2.61 ± 0.26 Hz, Z = -2.18 and p = 0.03).

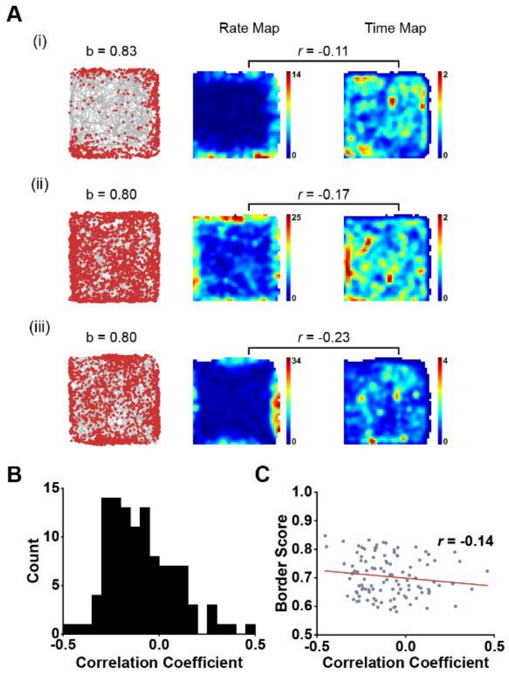

Supplementary Fig. S5. Spatial representations of mPFC border cells are not artifacts from more occupancy at geometric boundaries.

(**A**) Correlation between firing rate map and occupancy time map for three representative mPFC border cells. Left, trajectory (grey line) with superimposed spike locations (red dots) with border score labeled at the top of the panels; Middle, firing rate maps; Middle, firing rate maps; Right, occupancy time maps. Correlation coefficient (*r*) was labeled at the top of the panels. (**B**) Histogram showing the distribution of correlation coefficients between firing rate maps and occupancy time maps for all identified border cells (n = 110). (**C**) The relationship between border score and correlation coefficient between firing rate map and occupancy time map with red line being a linear fit (Pearson's r = -0.14, p = 0.15).

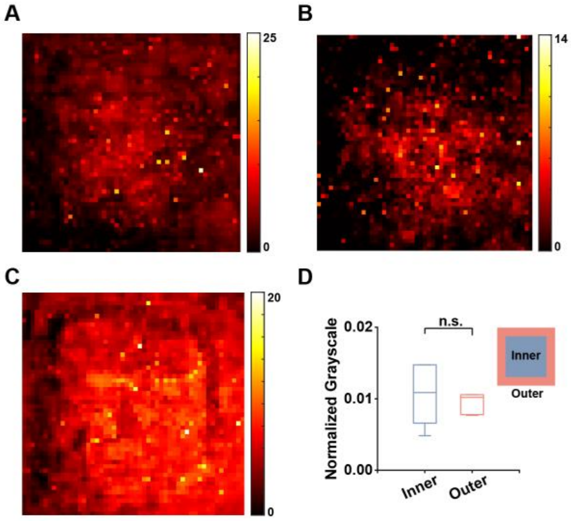

Supplementary Fig. S6. Distribution of food pellets in the running arena.

(**A**-**C**) Heat maps of the distribution of crumbed food pellets (~3-6 g) randomly thrown into the running arena without the rats in the running arena from three separate sessions. (**D**) Comparison of normalized grayscale value between the inner zone and outer zone (n = 6, two-sided Wilcoxon signed rank test, Z = -0.31 and p = 0.75).

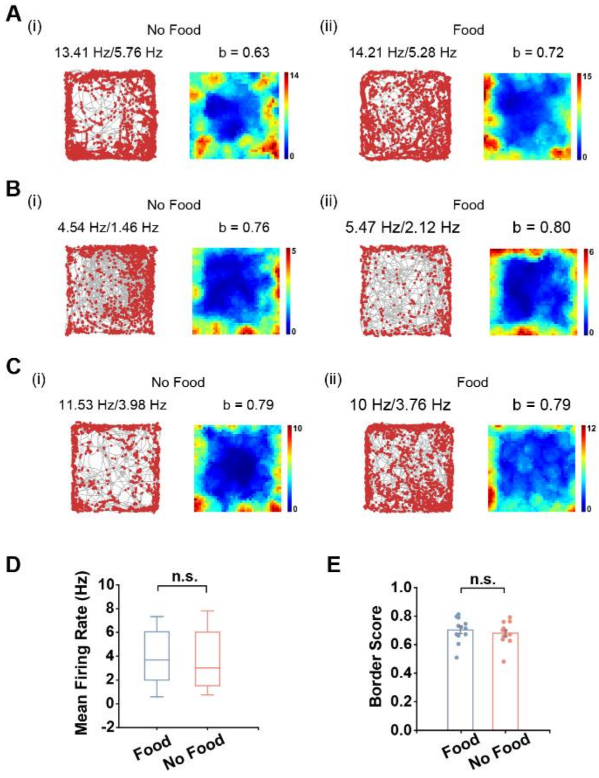

Supplementary Fig. S7. Spatial responses of mPFC border cells are preserved in the absence or presence of reward.

(**A**-**C**) Spatial responses in the absence or presence of food for three mPFC border cells. (**D**) Comparison of mean firing rate (n = 12, two-sided Wilcoxon signed rank test, Z = -0.47 and p = 0.64). (**E**) Comparison of border score (n = 12, two-sided Wilcoxon signed rank test, Z = -1.41 and p = 0.16).

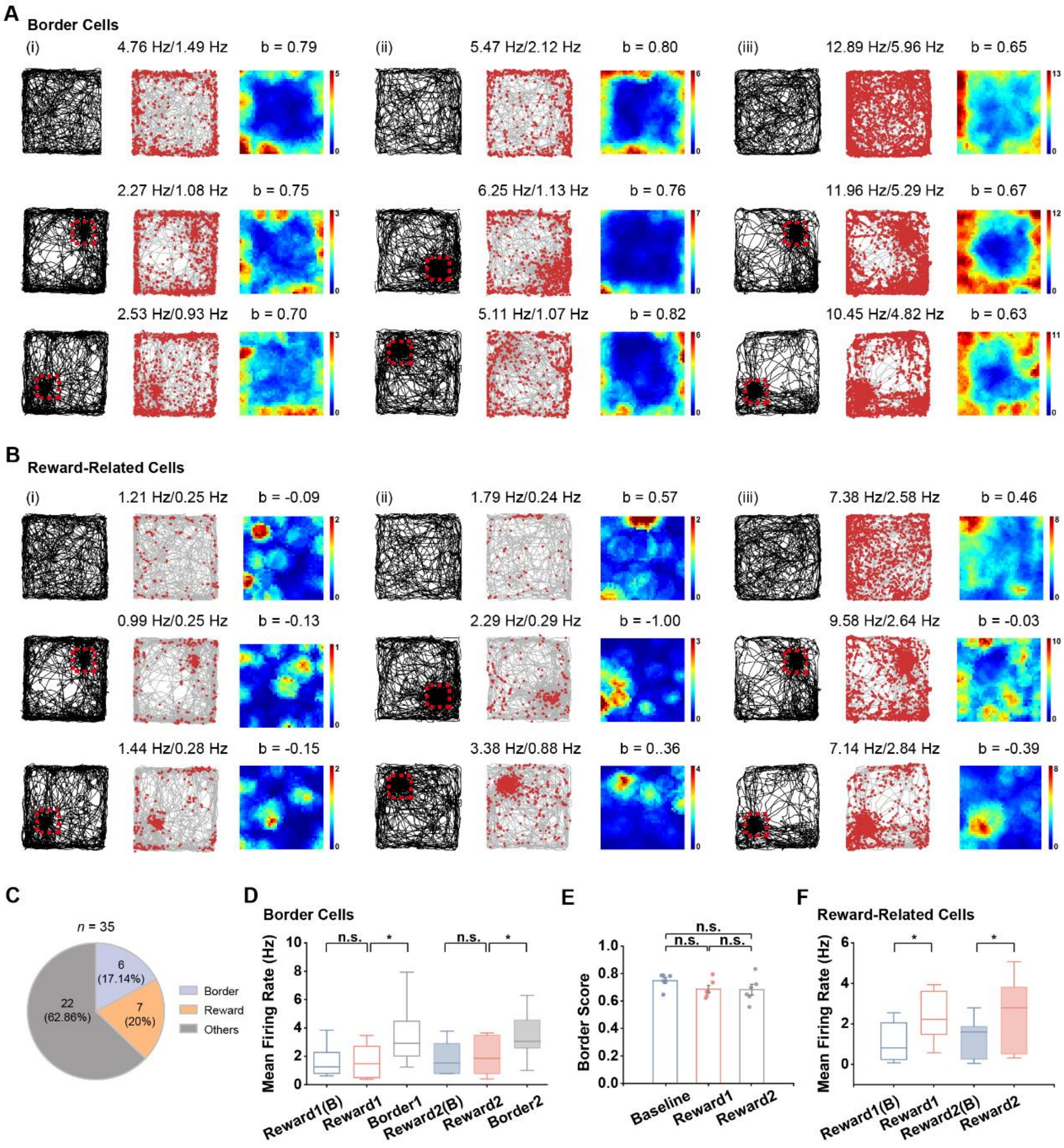

Supplementary Fig. S8. Spatial responses of mPFC cells to the delivery of reward to a fixed location in the running arena.

(**A**) Spatial responses in one recording session with randomly scattered food (upper panels) or two recording sessions with two different fixed locations of food delivery (middle and bottom panels) for three mPFC border cells. (**B**) Same as in (**A**) except for three rewarded-related cells. (**C**) Pie chart showing the composition of three types of cells (*n* = 35): border cells, reward-related cells and other cells. A cell was defined as a reward-related cell if the firing rate in the reward zone (25 cm × 25 cm) during the recording session with fixed location of reward was at least 1.5 times higher than that in the same area during the baseline recording session. (**D**) Comparison of the mean firing rate of the two reward areas (Reward1 and Reward2), the corresponding same

area in the first baseline session [Reward1(B) and Reward2(B)] and along the borders in the two recording sessions with fixed location of food delivery (Border1 and Border2) (n = 6, two-sided Wilcoxon signed rank test, Reward1(B) versus Reward1, Z = -0.11 and p = 0.92; Reward1 versus Border1, Z = -2.20 and p = 0.03; Reward2(B) versus Reward2, Z = -0.11 and p = 0.92; Reward2 versus Border2, Z = -2.20 and p = 0.03). (**E**) Comparison of border score across three recording sessions (n = 6, two-sided Wilcoxon signed rank test, Baseline versus Reward1, Z = -1.57 and p = 0.12; Reward1 versus Reward2, Z = -0.31 and p = 0.75; Baseline versus Reward2, Z = -1.57 and p = 0.12). (**F**) Comparison of mean firing rate in the reward area for reward-related cells in the baseline and reward sessions (n = 7, two-sided Wilcoxon signed rank test, both Z = -2.37 and p = 0.02).

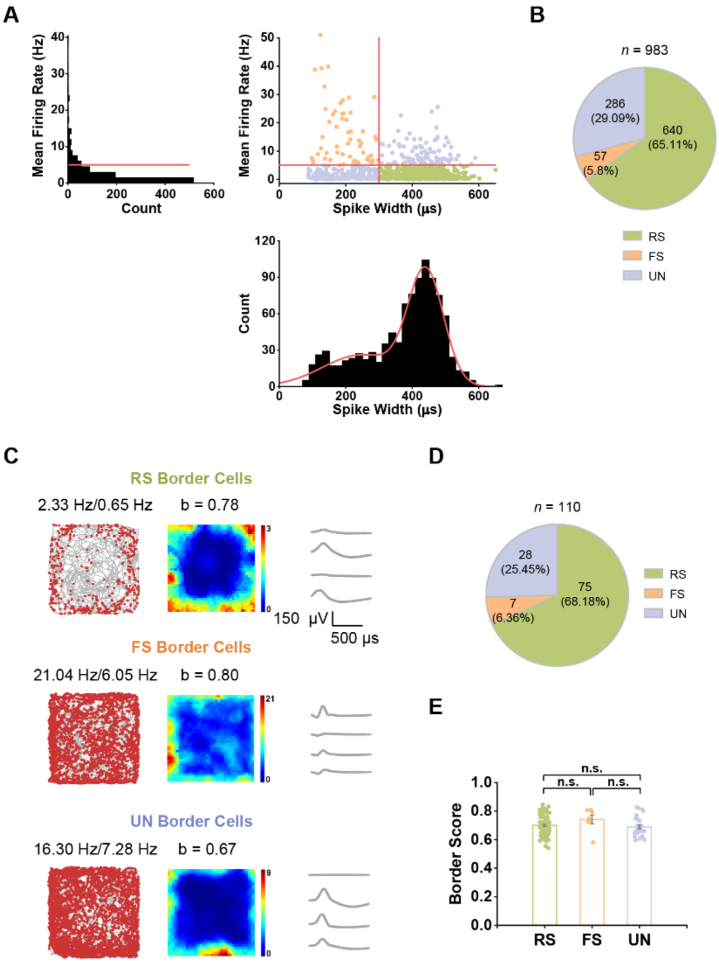

Supplementary Fig. S9. Cell type classification of mPFC border cells.

(**A**) Cell classification based on the waveform and mean firing rate. Top right, scatter plot of mean firing rate versus peak-to-trough waveform duration for the population of somatosensory cells. Left, histogram of mean firing rates for the whole population. Bottom, histogram of peak-to-trough spike widths for the whole population. A mixture of two Gaussians (red trace) was fitted and a local minimum (300 μs) was used as the classification threshold. (**B**) Pie chart showing the proportion of regular-spiking (RS), fast-spiking (FS) and unclassified (UN) cells. (**C**) Representative regular-spiking (RS), fast-spiking (FS) and unclassified border cells from mPFC. Notations and symbols are similar to those in **Fig. 1**. (**D**) Pie chart showing the cell-type classification of mPFC border cells. (**E**) The border score was comparable among the three types of border cells.

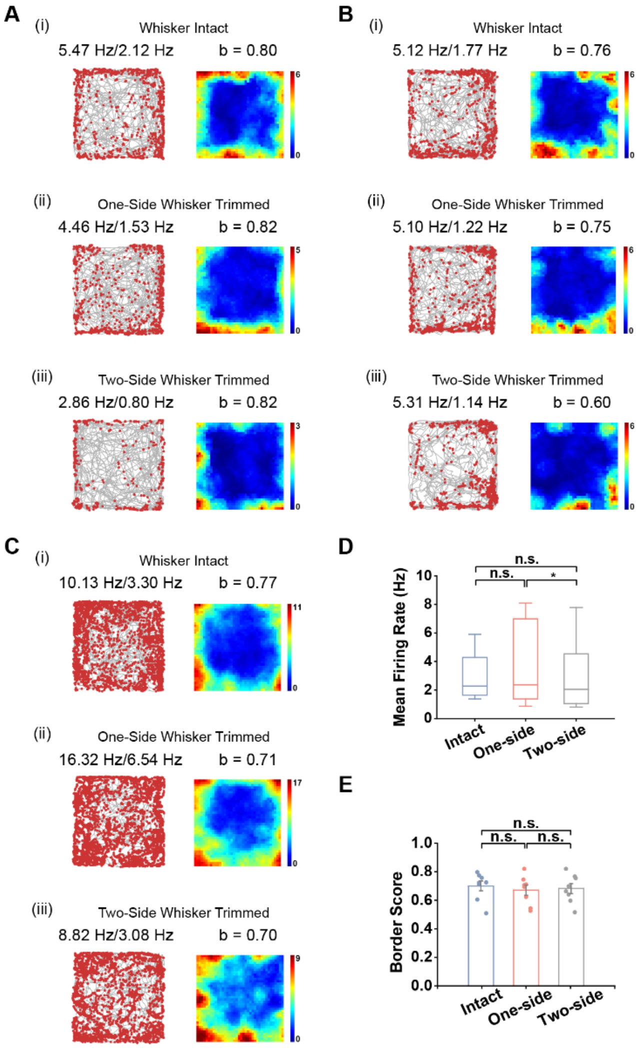

Supplementary Fig. S10. Spatial responses of mPFC border cells preserve after whisker removal.

(**A**-**C**) Spatial responses before (upper panels), after one-side whisker trimming (middle panels) and after two-side whisker trimming (bottom panels) for three mPFC border cells. (**D**) Comparison of mean firing rate (n = 8, two-sided Wilcoxon signed rank test, intact versus one-side whisker trimming, Z = -1.12 and p = 0.26; one-side versus two-side whisker trimming, Z = -2.10 and p = 0.04; intact versus one-side whisker trimming, Z = -0.14 and p = 0.89). (**E**) Comparison of border score (same test, intact versus one-side whisker trimming, Z = -0.84 and p = 0.40; one-side versus two-side whisker trimming, Z = -0.17 and p = 0.87; intact versus one-side whisker trimming, Z = -0.42 and p = 0.67).

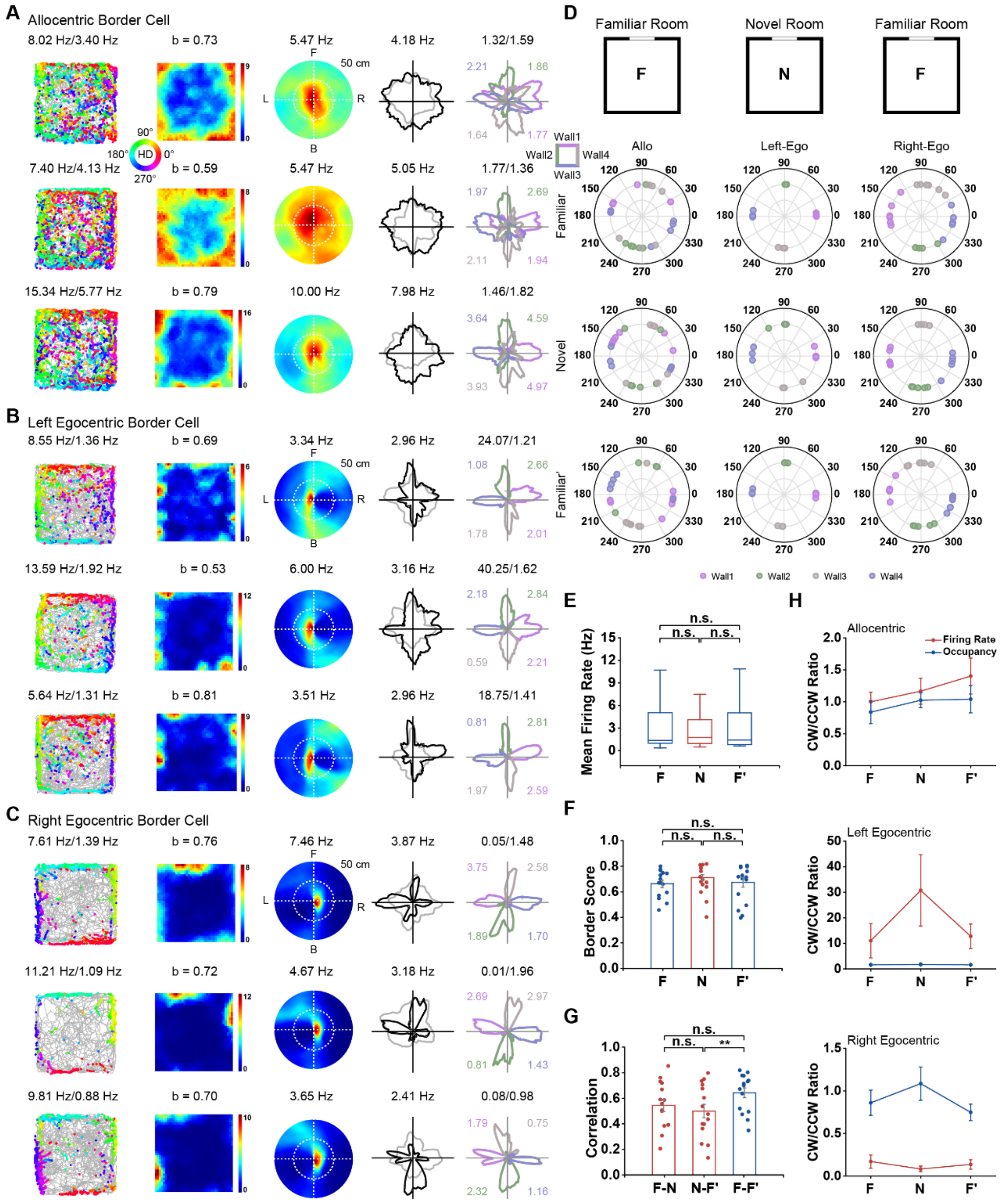

Supplementary Fig. S11. mPFC border cells remain stable in novel environments.

(**A**-**C**) Spatial responses of representative allocentric (**A**), left egocentric (**B**) and right egocentric (**C**) border cells, respectively, in the familiar (F), novel (N) and back in the familiar (F') environment. Notations and symbols are similar to those in **Fig. 2**. (**D**) Polar plot showing the distribution of peak direction of head-direction tuning for four color-coded walls. (**E**) Mean firing rate showed no significant change in the novel environment (n = 15, two-sided Wilcoxon signed rank test, F-N, Z = -0.51 and p = 0.61; N-F', Z = -0.63 and p = 0.53; F-F', Z = -0.23 and p = 0.82). (**F**) Border score was not

statically different between distinct rooms (same test, F-N, Z = 0.80 and p = 0.43; N-F', Z = -0.34 and p = 0.73; F-F', Z = -0.23 and p = 0.82). (**G**) The spatial correlation between firing rate maps in novel and familiar rooms was stable (same test, F-N, Z = -1.42 and p = 0.16; N-F', Z = -2.84 and p = 0.005; F-F', Z = -1.87 and p = 0.06). (**H**) Left and right egocentric border cells maintained CW/CCW ratio of firing rate in the novel environments, while the CW/CCW ratio of firing rate for allocentric border cells fluctuated in the range of 0.5 to 2.

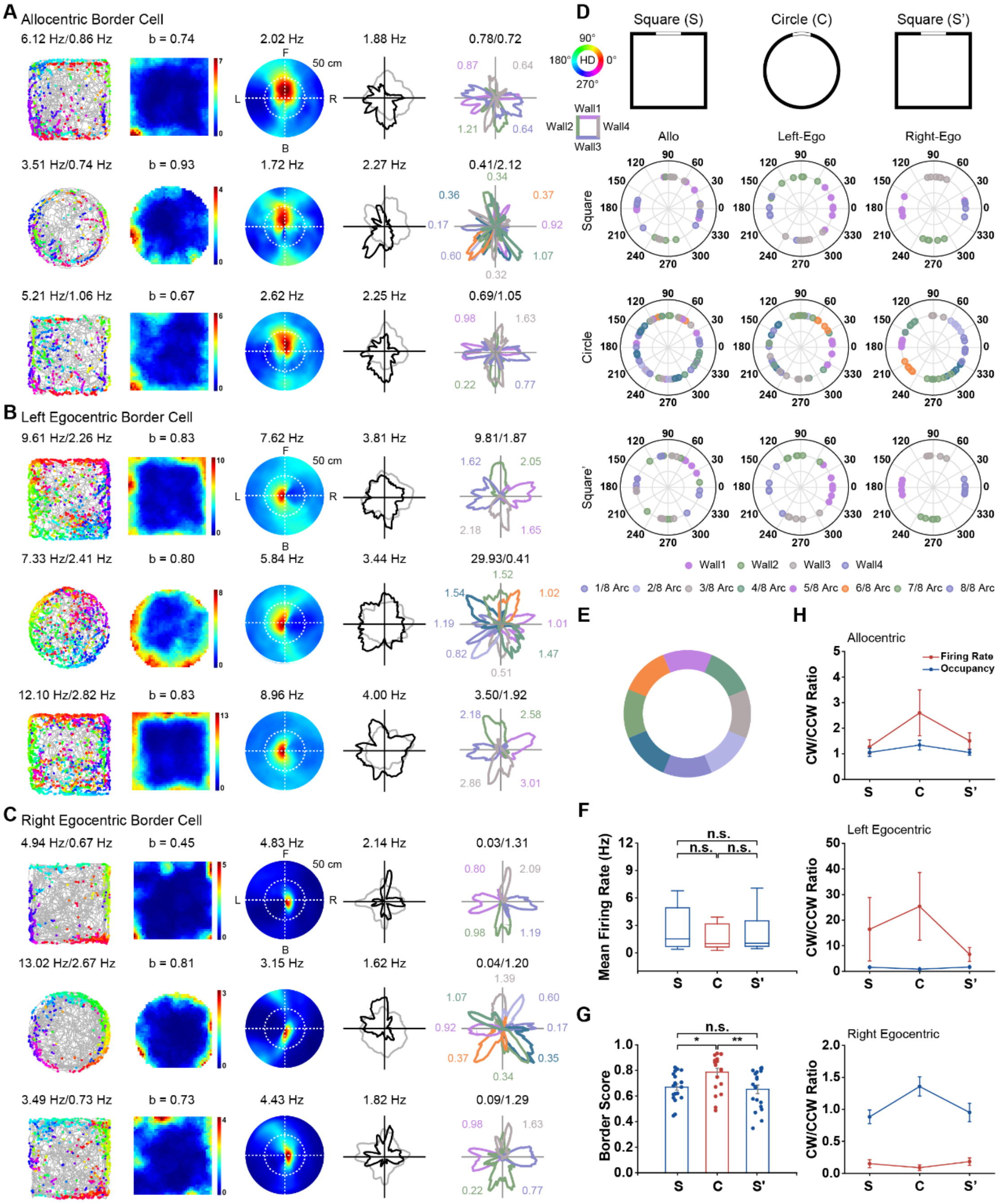

Supplementary Fig. S12. mPFC border cells preserve spatial firing patterns in circular environments.

(**A-C**) Spatial responses of representative allocentric (**A**), left egocentric (**B**) and right egocentric (**C**) border cells, respectively, in the square (S), circle (C) and back in the square (S') enclosures. (**D**) Top, diagram of the experimental procedure with the square, circular and square conditions. Bottom, summary of peak directions in eight arcs of circular boxes for allocentric, left egocentric and right egocentric border cells. The peak directions gradually changed and left and right egocentric border cells exhibited opposite directional tuning in the same arc. (**E**) Color-coded eight arcs of the

circular boundary. (**F**) The mean firing rate showed no significant change between the square and circle enclosures (n = 20, two-sided Wilcoxon signed rank test, S-C, Z = 1.01 and p = 0.31; C-S', Z = -0.28 and p = 0.78; S-S', Z = -0.64 and p = 0.53). (**G**) The border score was not statistically different between distinct geometric shapes (same test, S-C, Z = -2.33 and p = 0.20; C-S', Z = -2.65 and p = 0.008; S-S', Z = -0.67 and p = 0.50). (**H**) The CW/CCW firing rate ratio was maintained across environments of distinct geometric shapes. Curves in blue represent time_cw/ccw and curves in red represent fr_cw/ccw.

Supplementary References

1. Zhang S-J*, et al.* (2013) Optogenetic dissection of entorhinal-hippocampal functional connectivity. *Science* 340(6128):1232627.
2. Long X, Deng B, Cai J, Chen ZS, & Zhang S-J (2021) Egocentric asymmetric coding in sensory cortical border cells. *bioRxiv*.
3. Long X & Zhang S-J (2021) A novel somatosensory spatial navigation system outside the hippocampal formation. *Cell research* 31(6):649-663.
4. Frank LM, Brown EN, & Wilson MA (2001) A comparison of the firing properties of putative excitatory and inhibitory neurons from ca1 and the entorhinal cortex. *Journal of neurophysiology* 86(4):2029-2040.
5. Peyrache A*, et al.* (2012) Spatiotemporal dynamics of neocortical excitation and inhibition during human sleep. *Proceedings of the National Academy of Sciences of the United States of America* 109(5):1731-1736.
6. Long X*, et al.* (2022) Sharp tuning of head direction and angular head velocity cells in the somatosensory cortex. *Advanced science* 9(14):e2200020.
7. Mruczek RE & Sheinberg DL (2012) Stimulus selectivity and response latency in putative inhibitory and excitatory neurons of the primate inferior temporal cortex. *Journal of neurophysiology* 108(10):2725-2736.
8. Boccara CN*, et al.* (2010) Grid cells in pre- and parasubiculum. *Nature Neuroscience* 13(8):987-994.
9. Solstad T, Boccara CN, Kropff E, Moser MB, & Moser EI (2008) Representation of geometric borders in the entorhinal cortex. *Science* 322(5909):1865-1868.
10. Zhang SJ*, et al.* (2013) Optogenetic dissection of entorhinal-hippocampal functional connectivity. *Science* 340(6128):1232627.
11. Sargolini F*, et al.* (2006) Conjunctive representation of position, direction, and velocity in entorhinal cortex. *Science* 312(5774):758-762.
12. Alexander AS*, et al.* (2020) Egocentric boundary vector tuning of the retrosplenial cortex. *Science Advances* 6(8):eaaz2322.
13. Hinman JR, Chapman GW, & Hasselmo ME (2019) Neuronal representation of environmental boundaries in egocentric coordinates. *Nature Communications* 10(1):2772.

14. Paxinos G & Watson C (2007) *The rat brain in stereotaxic coordinates* (Elsevier, Amsterdam).
